# Target of rapamycin controls hyphal growth and pathogenicity through FoTIP4 in *Fusarium oxysporum*

**DOI:** 10.1111/mpp.13108

**Published:** 2021-07-20

**Authors:** Linxuan Li, Tingting Zhu, Yun Song, Xiumei Luo, Raju Datla, Maozhi Ren

**Affiliations:** ^1^ Institute of Urban Agriculture Chinese Academy of Agricultural Sciences Chengdu National Agricultural Science and Technology Center Chengdu China; ^2^ Zhengzhou Research Base State Key Laboratory of Cotton Biology Zhengzhou University Zhengzhou China; ^3^ School of Life Sciences Liaocheng University Liaocheng China; ^4^ Global Institute for Food Security in Saskatoon University of Saskatchewan Saskatoon Canada

**Keywords:** FoTOR1 interacting protein 4, *Fusarium oxysporum*, hyphal growth, pathogenicity, target of rapamycin

## Abstract

*Fusarium oxysporum* is the causal agent of the devastating Fusarium wilt by invading and colonizing the vascular system in various plants, resulting in substantial economic losses worldwide. Target of rapamycin (TOR) is a central regulator that controls intracellular metabolism, cell growth, and stress responses in eukaryotes, but little is known about TOR signalling in *F. oxysporum*. In this study, we identified conserved FoTOR signalling pathway components including FoTORC1 and FoTORC2. Pharmacological assays showed that *F. oxysporum* is hypersensitive to rapamycin in the presence of *FoFKBP12* while the deletion mutant strain Δ*Fofkbp12* is insensitive to rapamycin. Transcriptomic data indicated that FoTOR signalling controls multiple metabolic processes including ribosome biogenesis and cell wall‐degrading enzymes (CWDEs). Genetic analysis revealed that FoTOR1 interacting protein 4 (FoTIP4) acts as a new component of FoTOR signalling to regulate hyphal growth and pathogenicity of *F. oxysporum*. Importantly, transcript levels of genes associated with ribosome biogenesis and CWDEs were dramatically downregulated in the Δ*Fotip4* mutant strain. Electrophoretic mobility shift assays showed that FoTIP4 can bind to the promoters of ribosome biogenesis‐ and CWDE‐related genes to positively regulate the expression of these genes. These results suggest that FoTOR signalling plays central roles in regulating hyphal growth and pathogenicity of *F. oxysporum* and provide new insights into FoTOR1 as a target for controlling and preventing Fusarium wilt in plants.

## Graphical abstract

Genetic analysis revealed that FoTOR1 interacting protein 4 (FoTIP4) acts as a new component of FoTOR signalling to regulate hyphal growth and pathogenicity of *Fusarium oxysporum*.

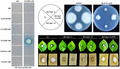

## 1 INTRODUCTION

The fungus *Fusarium oxysporum*, a hemibiotrophic and soilborne species with more than 100 formae speciales, is the causal agent of Fusarium wilt disease, which affects many important agricultural crops worldwide, such as cotton, banana, tomato, and potato (Garcia Bayona et al., 2011; Husaini et al., 2018; Ozbay & Newman, 2004; Ploetz, 2015; Raza et al., 2016; Schmidt et al., 2016), resulting in economic losses. *F. oxysporum* is the major pathogen in various Fusarium wilt diseases and was ranked fifth in a survey of the top 10 fungal plant pathogens (Dean et al., 2012), and one of the most difficult plant diseases to control because the spores of *F. oxysporum* can survive in soil for over 10 years (Husaini et al., 2018). The proximity of roots induces the dormant chlamydospores to germinate and initiate infection. Elongated hyphae start to adhere to the plant root surface and penetrate the roots through wounds or root tips. Ultimately, invasive hyphae reach the xylem vessels and proliferate, causing disease (Berrocallobo & Molina, 2008; Di et al., 2003). In the process of phytopathogenic fungi infecting the host, the plant cell wall is a major physical barrier that phytopathogenic fungi must overcome by producing a variety of cell wall‐degrading enzymes (CWDEs) that allow fungi to invade host tissues through the degradation of plant cell wall components. The pathogen *F. oxysporum* secretes various CWDEs, such as pectinase, cellulase, and β‐glucosidase, to degrade the plant cell wall (Calero‐Nieto et al., 2007; Jonkers, 2009; Ospina‐Giraldo et al., 2003). As a result, pectin can obstruct the vasculature in plants, thereby preventing water absorption, resulting in plant wilt and death (King et al., 2011); free monosaccharide and oligosaccharides that originate from the plant cell wall are used for fungal growth and reproduction (Gibson et al., 2011).

Target of rapamycin (TOR) is an evolutionarily conserved Ser/Thr protein kinase in eukaryotes. It is well known that the TOR signalling pathway regulates cell growth and proliferation in response to nutrients, energy, and stresses (De Virgilio & Loewith, 2006; Dobrenel et al., 2016; Yuan et al., 2013). Two *TOR* genes were first identified by screening rapamycin (RAP)‐insensitive mutants in budding yeast (*Saccharomyces cerevisiae*) (Heitman et al., 1991b; Kunz et al., 1993). However, only a single *TOR* gene has been identified in *Arabidopsis thaliana*, most animals, and humans (Menand et al., 2002; Sabers et al., 1995). The TOR protein contains five conserved regions: HEAT repeats, a FAT domain, an FRB domain, a kinase domain, and an FATC domain (Schmelzle & Hall, 2000; Wullschleger et al., 2006). TOR forms two structurally and functionally distinct protein complexes: TOR complex 1 (TORC1) and TORC2 (Loewith et al., 2002). Among eukaryotic species, TORC1 contains the TORC1 regulatory subunits KOG1 (known as RAPTOR in mammals) and Lethal with SEC13 protein 8 (LST8), which regulates cell growth and metabolism in response to nutrient and energy requirements (Wang & Proud, 2009). TORC2 possesses two TORC2‐specific subunits, AVO3 (known as RICTOR in mammals) and AVO1 (known as SIN1 in mammals), which controls spatial cell growth and survival by regulating cytoskeletal structure and polarity (De Virgilio & Loewith, 2006; Wullschleger et al., 2006). Despite functional importance, little is known about the TOR signalling pathway in *F. oxysporum*.

In rapidly growing cells, ribosome biogenesis is a major energy‐consuming process that accounts for a significant proportion of total transcriptional output (Warner, 1999). The regulation of ribosome biogenesis occurs primarily at the transcriptional level and involves all three nuclear RNA polymerases (Iadevaia et al., 2014; Martin et al., 2006). TORC1 positively regulates several steps in ribosome biogenesis, including ribosomal RNA transcription and the synthesis of ribosomal proteins and other components required for ribosome assembly and biogenesis (Ben‐Sahra et al., 2013; Chauvin et al., 2014; Tsang et al., 2010). TORC1 interacts directly with Sfp1 and phosphorylates Sfp1 to regulate ribosome biogenesis (Lempiainen et al., 2009). Sfp1 is a transcriptional activator of ribosome biogenesis genes (Fingerman et al., 2003; Marion et al., 2004). Under optimal growth conditions, Sfp1 is phosphorylated by TORC1 and then binds to the promoters of ribosome biogenesis genes to promote their expression in the nucleus. By contrast, nutrient depletion results in its relocalization from the nucleus to the cytoplasm in yeast (Lempiainen et al., 2009). The AGC‐family kinase SCH9 (known as S6K in mammals) is another downstream regulatory factor of TORC1. TORC1 phosphorylates and activates SCH9 to regulate ribosome protein synthesis (Chauvin et al., 2014; Iadevaia et al., 2014; Magnuson et al., 2012).

RAP is a macrolide immunosuppressant produced by *Streptomyces hygroscopicus*. It mimics nutrient limitation to arrest cell growth and proliferation. RAP specifically binds to FK506 binding protein of 12 kDa (FKBP12), which interacts with the FRB domain of TOR to form a ternary complex (Heitman et al., 1991b; Loewith et al., 2002). The resulting complex prevents TOR from associating with its scaffold protein RAPTOR and phosphorylating its substrate proteins (Aylett et al., 2016; Hara et al., 2002), which hinders TOR protein activity and results in irreversible arrest of the cell cycle at the G1 phase (Heitman et al., 1991a). RAP can inhibit TORC1, but TORC2 is insensitive to RAP (Loewith et al., 2002). The TORC2‐specific subunit RICTOR plays indispensable roles in TORC2 function (Gaubitz et al., 2016; Wullschleger et al., 2005). A recent crosslinking study of TORC2 in budding yeast demonstrated that the C‐terminus of RICTOR occupies the FRB domain of TOR kinase, preventing the RAP‐FKBP12 complex from binding to the FRB domain of TOR kinase in TORC2, which makes TORC2 insensitive to RAP (Gaubitz et al., 2015). Recent studies have also revealed that treatment with ATP‐competitive TOR protein kinase inhibitors, including Torin1, Torin2, Ku‐0063794 (KU), and AZD‐8055 (AZD), can result in different effects on both TORC1 and TORC2 than RAP treatment (Chresta et al., 2010; Garcia‐Martinez et al., 2009), as TOR is directly and specifically targeted by the ATP‐binding pocket of the TOR kinase domain, suppressing the functions of both TORC1 and TORC2 complexes (Benjamin et al., 2011).

Potato is a major staple food worldwide, but potato Fusarium wilt and dry rot diseases caused by *F*. *oxysporum* are global challenges for potato production. The TOR signalling pathway plays critical roles in regulating mycelial growth and virulence in fungi, and inhibition of TOR activity significantly reduces mycelial growth and pathogenicity in *Botrytis cinerea*, *Verticillium dahliae*, and *Fusarium graminearum* (Li et al., 2019; Xiong et al., 2019; Yu et al., 2014). In this study, we functionally characterized the conserved FoTOR signalling pathway in the regulation of mycelial growth and pathogenicity of *F*. *oxysporum*. RNA sequencing (RNA‐seq) analysis showed that FoTOR signalling plays vital roles in multiple intracellular and extracellular processes, including ribosome biogenesis and cell wall degradation. Additionally, FoTOR1 interacting protein 4 (FoTIP4), a new component of the FoTOR signalling pathway, was identified and characterized. Our findings also show that FoTIP4 plays an important role in the regulation of ribosome biogenesis and cell wall degradation in *F. oxysporum*. Our results suggest that FoTOR1 may serve as a promising target for controlling and preventing Fusarium wilt caused by *F. oxysporum* in plants.

## 2 RESULTS

### 2.1 The TOR signalling pathway is conserved in *F. oxysporum*

**FIGURE 1:**
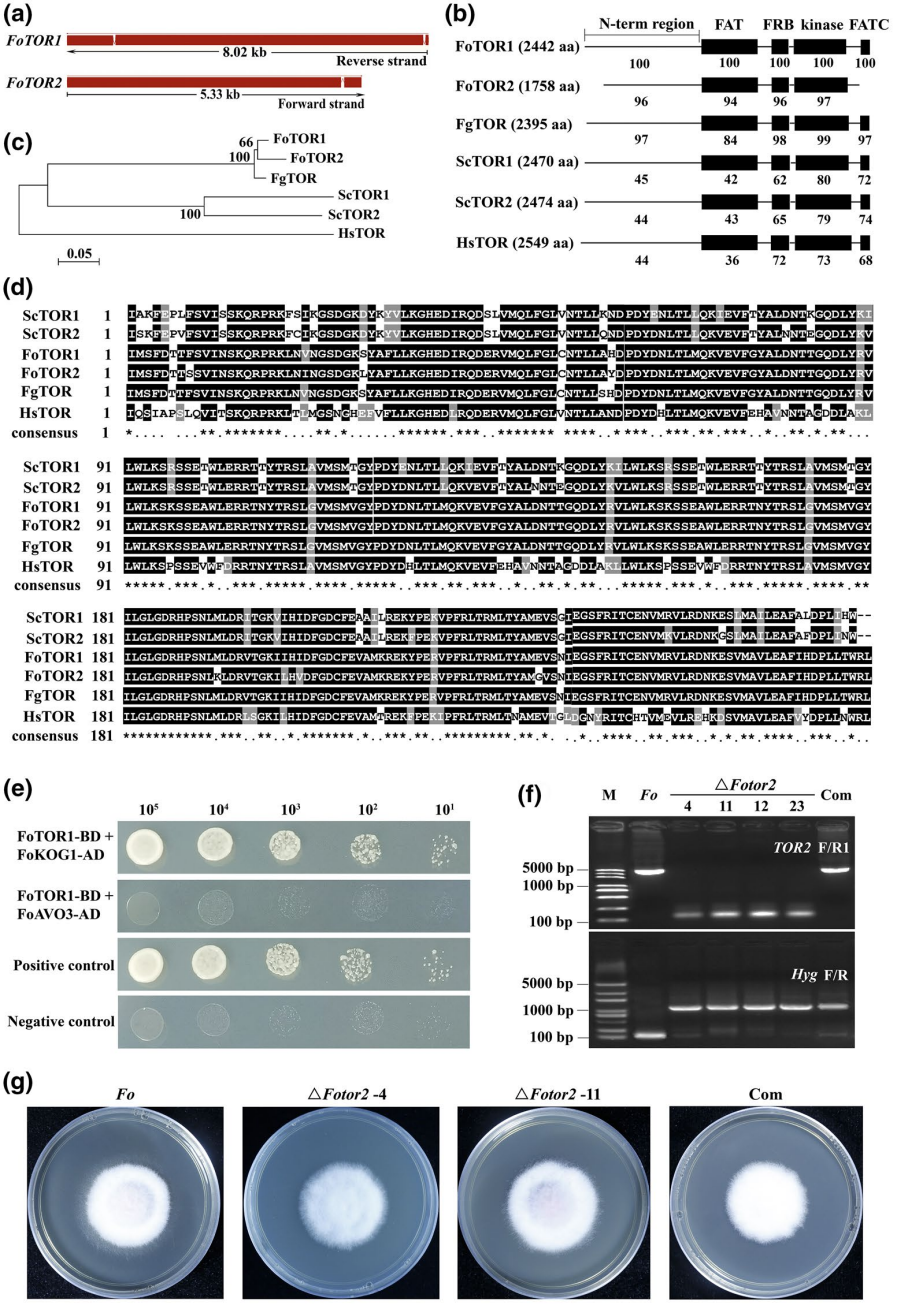
Structural analysis and functional characterization of FoTORs in *Fusarium oxysporum*. (a) The genome structure of *FoTORs*. Red represents exons and white rectangles indicate introns. (b) The conserved functional domains of FoTOR proteins. Conserved domains of FoTOR proteins are compared with those from other organisms. Each value indicates the percentage of identity with the corresponding domain sequences of FoTOR1. The number in parentheses represents the number of amino acids. aa, amino acids. (c) Phylogenetic relationship between the FoTOR proteins and homologues of *Saccharomyces cerevisiae* (*Sc*), *Homo sapiens* (*Hs*), and *Fusarium graminearum* (*Fg*). The phylogenetic tree was generated with MEGA 4.0 using the neighbour‐joining method. (d) Comparison of amino acid sequences of the kinase domains of FoTOR proteins with those from other representative organisms, including *Sc*, *Hs*, and *Fg*. (e) Yeast two‐hybrid analysis of the interaction between FoTOR1 and FoKOG1 or FoAVO3. Serial dilutions of yeast cells (cells/ml) transformed with the bait and prey constructs were assayed for growth on SD−His−Leu−Trp−Ade medium at 28 °C for 3 days. (f) Mutant generation of *FoTOR2* in *F. oxysporum*. Gel electrophoresis of the *TOR2* gene and the *Hyg* cassette. The *TOR2* gene and the *Hyg* cassette were amplified from wildtype *F. oxysporum*, Δ*Fotor2* mutants, and complemented (Com) strains with *TOR2* F/R1 and *Hyg* F/R primers, respectively. *Fo*, *F. oxysporum*; Com, complemented. (g) The phenotype of *FoTOR2* deletion mutants (Δ*Fotor2*) was similar to that of the wildtype *F. oxysporum* strain in potato dextrose agar

In order to identify evolutionarily conserved TOR signalling pathway components in *F. oxysporum*, BLASTp analysis of the *F. oxysporum* f. sp. *lycopersici* genome database (http://fungi.ensembl.org/Fusarium_oxysporum/Info/Index?db=core) using *Schizosaccharomyces pombe* TOR signalling pathway components as reference was performed. We found that putative homologous gene sequences encoding key components of TORC1, including TOR, KOG1, and LST8, were present in the genome of *F. oxysporum*, and putative homologues encoding specific components of TORC2, such as AVO3 and AVO1, were also found in the *F. oxysporum* genome (Table 1). Interestingly, two *TOR* gene homologues were detected (*FOXG_18412* and *FOXG_15946*) in the *F. oxysporum* genome (Lopez‐Berges et al., 2010) (Figure 1a). Further analysis revealed that the FoTOR1 (FOXG_18412) protein showed similar conserved domains as in yeast and human, whereas the FATC domain was not detected in the FoTOR2 (FOXG_15946) protein (Figure 1b). Phylogenetic analysis and kinase domain sequence alignment with proteins from other representative organisms indicated that FoTOR1 and FoTOR2 are evolutionarily conserved (Figure 1c,d). Interestingly, there is only one *TOR* gene in some formae speciales of *F. oxysporum* (Table S1). We found TOR kinase expansion in eight out of 14 sequenced *F. oxysporum* strains, with six strains containing one copy, seven strains containing two copies, and one strain containing three copies. Phylogenetic analysis showed that the orthologous copies of the TOR kinase (indicated as Core TOR) form a monophyletic group and nine TOR paralogues (indicated as LS TOR) clustered together (Figure S1); this is in agreement with a previous study (DeIulio et al., 2018).

**TABLE 1:** Putative components of the TOR signalling pathway in *Fusarium oxysporum*

Protein name	*Schizosaccharomyces pombe*	*Saccharomyces cerevisiae*	*Fusarium oxysporum*	Identity (%)	Chr
Target of rapamycin (TOR)	TOR1	TOR1	FoTOR‐like1 (FOXG_18412)	49	8
	TOR2	TOR2	FoTOR‐like2 (FOXG_15946)	40	2
Regulatory associate protein of TOR (RAPTOR)	Mip1	KOG1	FoKOG1‐like (FOXG_03095)	48	8
Lethal with SEC‐13 protein 8 (LST8)	Pop3	LST8	FoLST8‐like (FOXG_05169)	63	7
FK506 binding protein 12 (FKBP12)	Fkh1	FPR1	FoFKBP12‐like (FOXG_08379)	59	2
Stress‐activated MAP kinase‐interacting protein 1 (SIN1)	Sin1	AVO1	FoAVO1‐like (FOXG_09442)	28	9
Rapamycin‐insensitive companion of TOR (RICTOR)	Ste20	AVO3	FoAVO3‐like (FOXG_11510)	33	10
Adhere voraciously to TOR2	–	AVO2	FoAVO2‐like (FOXG_02176)	29	5
Type 2A phosphatase‐associated protein 42 (TAP42)	Tap42	TAP42	FoTAP42‐like (FOXG_08258)	37	2
Ribosomal protein S6 kinase (S6K)	Sck1	SCH9	FoSCH9‐like (FOXG_00582)	40	1
Sfp1	Sfp1	Sfp1	FoTIP4 (FOXG_00980)	23	1
Ribosome protein small subunit 6 (RPS6)	Rps6‐1	RPS6A	–	–	–
	Rps6‐2	RPS6B	FoRPS6B‐like (FOXG_00951)	77	1
eIF2α kinase	Gcn2	GCN2	FoGCN2‐like (FOXG_07881)	33	4
AMP‐activated protein kinase alpha subunit (AMPKα)	Ssp2	SNF1	FoSNF1‐like (FOXG_05528)	42	7
Catalytic subunit of protein phosphatase 2A (PP2AC)	Ppa2	PPH21	FoPPH21‐like (FOXG_08241)	76	2
Type 2A phosphatase regulator TIP41	Tip41	TIP41	FoTIP41‐like (FOXG_01852)	33	5
Translation initiation factor 2 subunit alpha (eIF2α)	Tif211	eIF2α	FoeIF2α‐like (FOXG_08491)	60	2

To verify the interactions between TOR and other key components of the TOR signalling pathway, we performed yeast two‐hybrid (Y2H) assays. We found that FoTOR1, rather than FoTOR2, interacted with FoKOG1 (Figures 1e and S2). Interestingly, neither FoTOR1 nor FoTOR2 interacted with FoAVO3 in Y2H assays, implying that there may be no functional TORC2 in *F. oxysporum*. To further determine FoTOR function, *FoTOR2* deletion mutants (Δ*Fotor2*) were created (Figure 1f). Morphological analyses showed that hyphal growth of the Δ*Fotor2* mutants was similar to that of the wildtype *F. oxysporum* strain (Figure 1g), indicating *FoTOR2* is dispensable for hyphal growth. However, all hygromycin‐resistant transformants of *FoTOR1* deletion mutants were ectopic mutants and we failed to get a null mutant. These results suggest that the deletion of *FoTOR1* in *F. oxysporum* may be lethal, indicating that FoTOR1 is an essential and central regulator of the FoTOR signalling pathway in *F. oxysporum*.

### 2.2 TOR inhibitors inhibit mycelial growth of *F. oxysporum*

**FIGURE 2:**
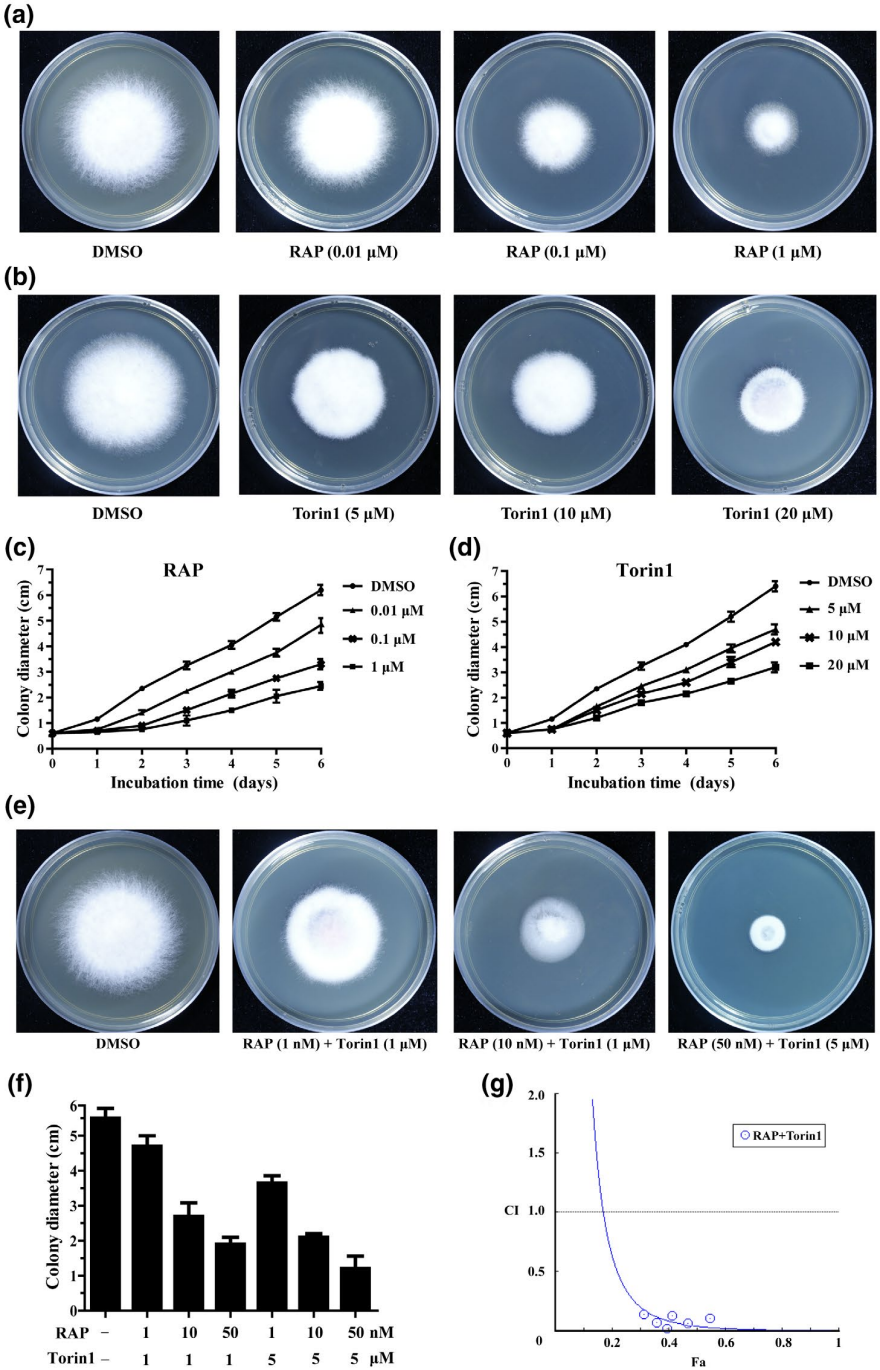
TOR inhibitors RAP and Torin1 inhibit hyphal growth in *Fusarium oxysporum*. (a) RAP can significantly inhibit hyphal growth of *F. oxysporum* in a dose‐dependent manner. Wildtype *F. oxysporum* was incubated on potato dextrose agar (PDA) plates containing different concentrations RAP for 6 days. (b) Torin1 partially inhibited hyphal growth of *F. oxysporum*. Conidia of *F. oxysporum* were incubated on PDA containing different concentrations of Torin1 for 6 days. (c) and (d) Colony diameter of wildtype *F. oxysporum* incubated on PDA with different concentrations of RAP or Torin1 from 0 to 6 days. The data are presented as the mean ± *SD* of *n* = 3 independent experiments. (e) and (f) Conidia of wildtype *F. oxysporum* were incubated on PDA plates with pairwise combinations of RAP and Torin1 for 6 days. (g) Fa–CI curve showing synergism (CI < 1) between RAP and Torin1

RAP is a well‐known TOR‐specific inhibitor. It specifically binds to FKBP12 to form a gain‐of‐function complex, which negatively regulates TOR kinase activity (Heitman et al., 1991b). To test whether RAP has an inhibitory effect on TOR in *F. oxysporum*, we first assayed the sensitivity of wildtype *F. oxysporum* to RAP. The growth inhibition of *F. oxysporum* was positively correlated with increasing concentrations of RAP (Figure 2a,c). Consistent with the results of a previous report (Lopez‐Berges et al., 2010), RAP inhibited hyphal growth and development in a dose‐dependent manner in *F. oxysporum*. Besides, the second‐generation TOR inhibitors Torin1, KU, and AZD were also used for the growth assay. Consistent with the observations in yeast (Atkin et al., 2014), Torin1 but not KU or AZD partially inhibited hyphal growth with a much higher half maximal inhibitory concentration (IC_50_) value (20 μM) than that of RAP (0.1 μM) (Figure 2b,d), indicating that ATP‐competitive inhibitors of TOR kinase have different effects on TOR in different species (Chresta et al., 2010; Garcia‐Martinez et al., 2009; Montane & Menand, 2013; Xiong et al., 2017). Furthermore, the germination rate and production of spores were reduced upon RAP and Torin1 treatment, and the relative transcript levels of sporulation‐related genes were significantly decreased upon FoTOR inhibition in *F*. *oxysporum* (Figure S3). In addition, we tested the sensitivity of the Δ*Fotor2* mutants to RAP and Torin1. The results showed that the growth of the Δ*Fotor2* mutant was similar to that of the wildtype *F. oxysporum* strain upon RAP and Torin1 treatment (Figure S4), indicating that TOR inhibitors inhibit mycelial growth of *F. oxysporum* in a FoTOR2‐independent manner. Combined treatment with RAP and Torin1 exerted more obvious growth inhibitory effects than treatment with RAP or Torin1 alone (Figure 2e). The IC_50_ value of a single drug (RAP 10 nM, Torin1 1 μM) was significantly reduced when *F. oxysporum* was subjected to combined treatment (Figure 2f), implying that potential synergistic effects can be generated by combining RAP with Torin1. Next, a computer‐simulated affected fraction (Fa)–combination index (CI) curve was assessed using CompuSyn software. A synergistic effect (CI < 1) was observed when hyphae were treated with a combination of RAP + Torin1 (Figure 2g). These results suggest that RAP and Torin1 inhibit hyphal growth by simultaneously targeting the TOR signalling pathway in *F. oxysporum*.

### 2.3 Deletion of *FoFKBP12* leads to insensitivity to RAP in *F. oxysporum*

**FIGURE 3:**
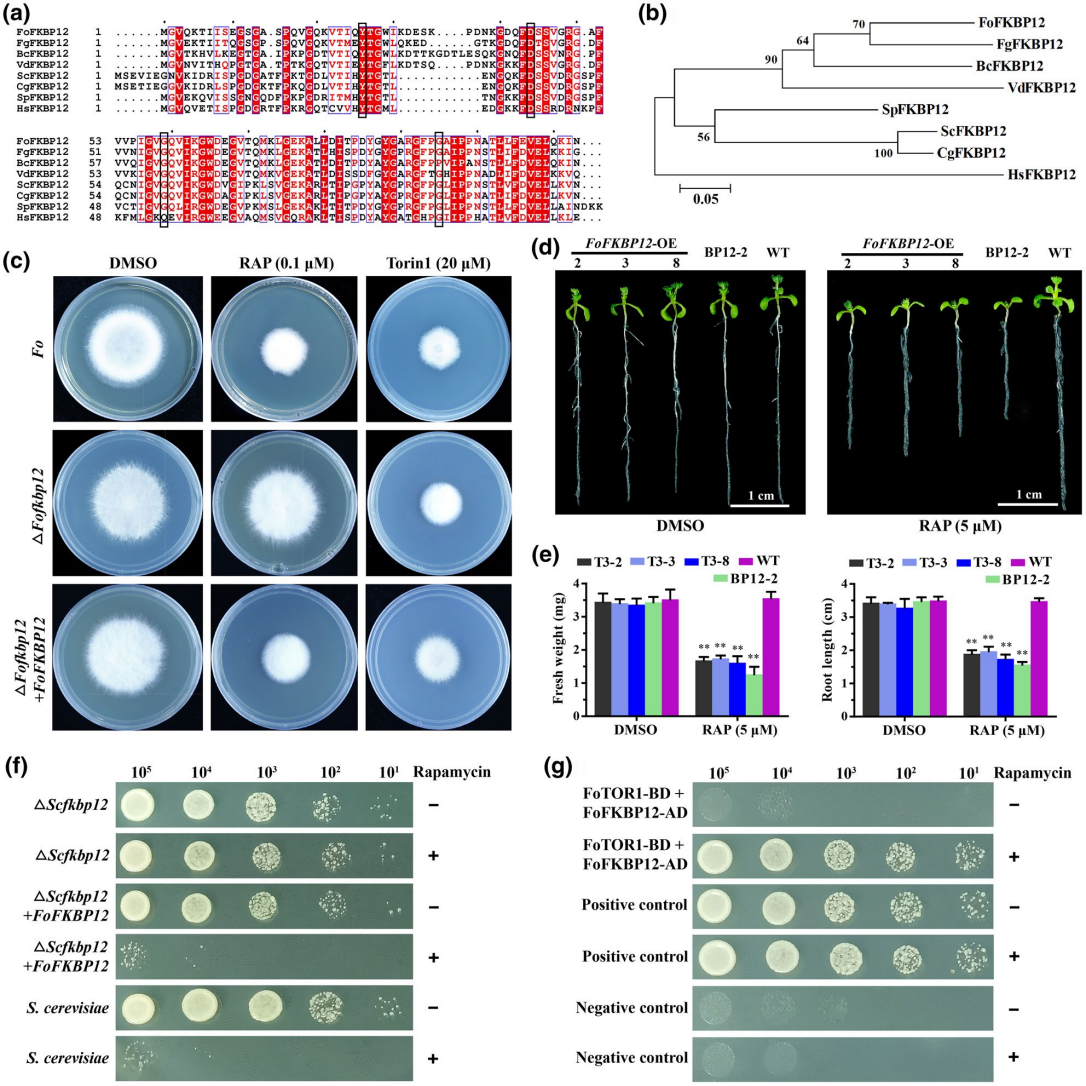
FoFKBP12 mediates inhibitory effects of RAP on FoTOR activity in *Fusarium oxysporum*. (a) Comparison of amino acid sequences of FKBP12 from *Saccharomyces cerevisiae* (*Sc*), *Homo sapiens* *(* *Hs* *)* *, Schizosaccharomyces pombe* (*Sp*)*, Candida glabrata* (*Cg*)*, Botrytis cinerea* (*Bc*)*, Verticillium dahliae* (*Vd*), *F. oxysporum* (*Fo*), and *Fusarium graminearum* (*Fg*). The black boxes indicate amino acids involved in ternary complex formation. (b) Phylogenetic relationship between FoFKBP12 protein and homologues from other organisms in (a). The phylogenetic tree was generated with MEGA 4.0 using the neighbour‐joining method (1,000 bootstrap replicates). (c) Deletion of *FoFKBP12* (Δ*Fofkbp12*) led to resistance to RAP in *F. oxysporum*. Hyphae of wildtype *F. oxysporum*, Δ*Fofkbp12*, and the complemented strain (Δ*Fofkbp12* + *FoFKBP12*) were incubated on medium containing DMSO, RAP, or Torin1 and grown for 5 days. (d) FoFKBP12 overexpression transgenic *Arabidopsis* lines were sensitive to RAP (5 μM). *FoFKBP12*, BP12‐2, and wildtype seeds were cultured on Murashige & Skoog (MS) plates containing DMSO or RAP for 10 days. Representative seedlings are shown. The experiment was repeated three times. Bar = 1 cm. (e) Fresh weight and root length of *FoFKBP12* overexpression transgenic *Arabidopsis* lines exposed to RAP (5 μM). Each column represents the average of 10 seedlings. The data are presented as the mean ± *SD* of *n* = 3 independent experiments. **p < 0.01 compared with wildtype plants (Student’s *t* test). (f) FoFKBP12 restored the sensitivity to RAP in the yeast FKBP12 mutant (Δ*Scfkbp12*). Strains growth on yeast‐peptone‐dextrose (YPD) medium with (+) or without (−) 1 μg/ml RAP at 28 °C for 3 days. (g) Yeast two‐hybrid analysis of the interaction between FoTOR1 and FoFKBP12. Serial dilutions of yeast Δ*Scfkbp12* mutant cells (cells/ml) transformed with the FoTOR1‐BD and FoFKBP12‐AD constructs were assayed for growth on SD−His−Leu−Trp−Ade medium with (+) or without (−) 1 μg/ml RAP at 28 °C for 3 days

We found that RAP effectively inhibited hyphal growth at a low concentration (Figure 2). It was previously reported that FKBP12 mediates the inhibitory effects of RAP on TOR (Heitman et al., 1991b). Therefore, we analysed the sequences of *FoFKBP12* in the *F. oxysporum* genome. A single copy of an *FKBP12* orthologue (*FOXG_08379*, named *FoFKBP12*) encoding a protein with 59% similarity to SpFKBP12 was found (Table 1). Sequence alignment and phylogenetic analysis showed that FoFKBP12 is evolutionarily conserved among species (Figure 3a,b). Amino acids known to be involved in the formation of the RAP inhibitory ternary complex were conserved in the FoFKBP12 sequence (Figure 3a). In order to test the ability of FoFKBP12 to bind RAP and TOR, we generated *FoFKBP12* deletion mutants (Δ*Fofkbp12*) by a homologous recombination gene deletion strategy (Figure S5a,b). Morphological analyses showed that hyphal growth of the mutant was similar to that of wildtype *F. oxysporum* on potato dextrose agar (PDA) (Figure 3c), suggesting that FoFKBP12 is dispensable for hyphal growth. The RAP sensitivity test showed that the Δ*Fofkbp12* mutant was insensitive to RAP, but the sensitivity to RAP was restored in the complemented strain (Δ*Fofkbp12* + *FoFKBP12*) (Figure 3c). As expected, the wildtype, Δ*Fofkbp12*, and Δ*Fofkbp12* + *FoFKBP12* lines showed the same growth phenotypes upon Torin1 treatment (Figure 3c), indicating that Torin1 inhibits mycelial growth of *F. oxysporum* in an FoFKBP12‐independent manner. A previous study showed that *Arabidopsis* has adapted an evolutionary mutation in the *FKBP12* gene, resulting in loss of its ability to bind RAP (Sormani et al., 2007). In order to further confirm the function of FoFKBP12, *FoFKBP12* overexpression *Arabidopsis* transgenic lines were generated. All *FoFKBP12* transgenic lines displayed sensitivity to RAP, which reflected shorter primary root length, smaller cotyledons, and lower fresh weight compared with wildtype *Arabidopsis* (Figure 3d,e), and this observation was consistent with observations in the *ScFKBP12* overexpression line (BP12‐2) in *Arabidopsis* (Ren et al., 2012). We tested the ability of FoFKBP12 to bind RAP in an *S. cerevisiae* (Y2HGold strain) *FKBP12* deletion mutant (Δ*Scfkbp12*). The wildtype yeast strain was sensitive to RAP while the Δ*Scfkbp12* mutant was RAP‐resistant. FoFKBP12 restored the sensitivity to RAP in the Δ*Scfkbp12* + *FoFKBP12* yeast strain (Figure 3f). We analysed the interaction between FoTOR1 and FoFKBP12 by Y2H assays. For each experiment, one pair of Y2H plasmids was cotransformed into the Δ*Scfkbp12* mutant strain. FoFFKBP12 was unable to interact with FoTOR1 without RAP treatment. By contrast, when the medium was supplemented with 1 μg/ml RAP, FoFKBP12 interacted strongly with FoTOR1 (Figure 3g). These results indicate that FoFKBP12 mediates the inhibitory effects of RAP on FoTOR activity in *F. oxysporum*.

### 2.4 FoTOR1 is a key regulator of ribosome biogenesis and CWDEs in *F. oxysporum*

**FIGURE 4:**
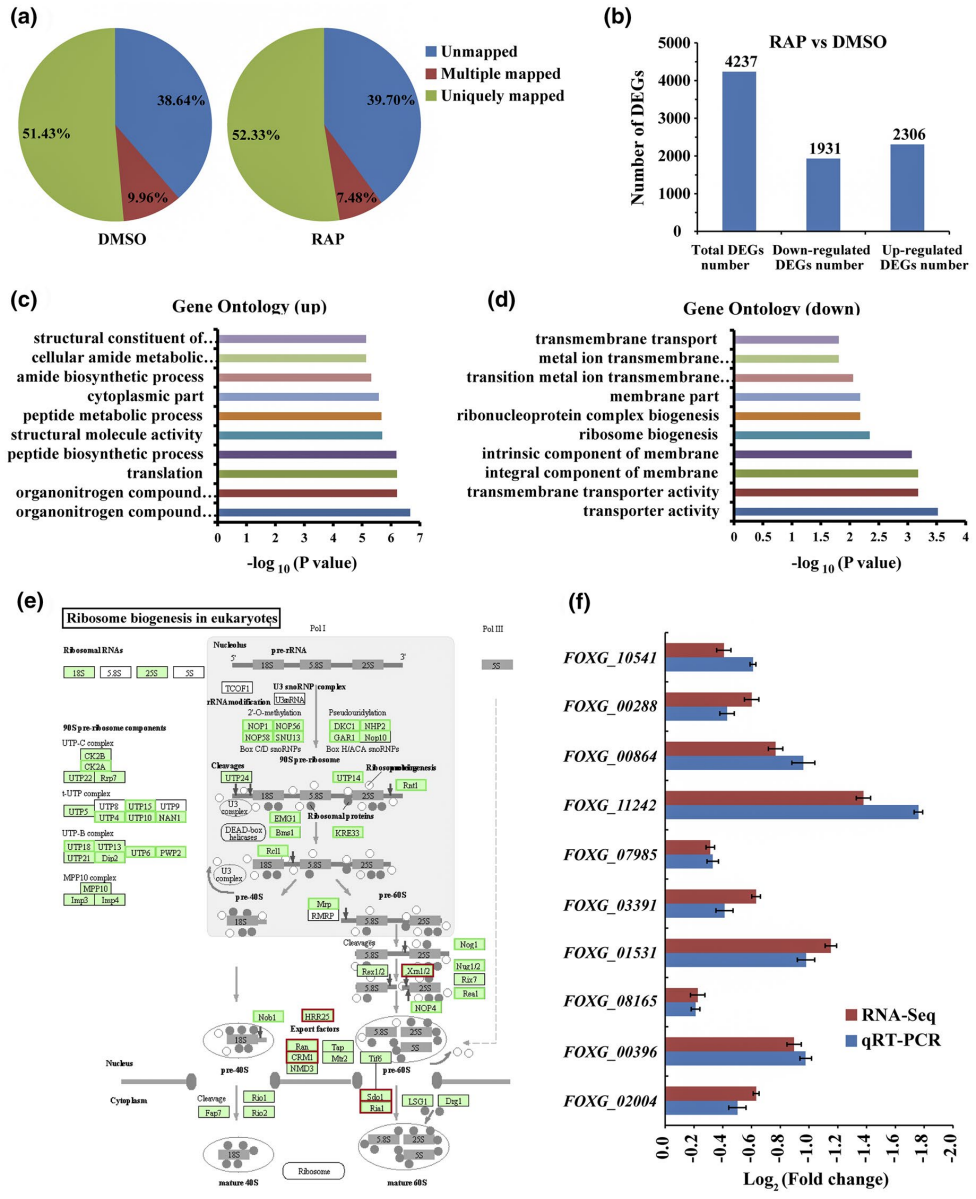
RNA‐seq analysis of *Fusarium oxysporum* mycelium treated with DMSO or RAP (*n* = 3 independent experiments). (a) Proportions of unmapped clean reads and clean reads mapped to multiple genes and mapped to unique genes. (b) Number of total, downregulated, and upregulated differentially expressed genes (DEGs). (c) and (d) Gene Ontology (GO) enrichment analysis of significantly upregulated and downregulated genes upon RAP treatment as identified by RNA‐seq. GO was ranked on the basis of significance. (e) Changes in expression of genes involved in ribosome biogenesis in eukaryotes. Green boxes indicate downregulated genes, red boxes indicate upregulated genes. (f) Quantitative reverse transcription PCR verification of the DEGs involved in ribosome biogenesis

The TOR signalling pathway integrates various extracellular and intracellular signals, such as growth factors, nutrients, energy, and other environmental cues, regulating multiple cellular processes, including ribosome biogenesis, protein synthesis, autophagy, and metabolic processes (De Virgilio & Loewith, 2006; Dobrenel et al., 2016; Kos‐Braun & Kos, 2017; Yuan et al., 2013). To further elucidate the function of the TOR signalling pathway in vegetative growth of *F. oxysporum*, a gene expression profile analysis was performed in *F. oxysporum* upon FoTOR inhibition with RAP treatment. Approximately 61% and 52% of the reads were mapped to the annotated *F. oxysporum* genome and the unigenes, respectively (Figure 4a). A total of 4,237 differentially expressed genes (DEGs) were found between the RAP‐ and DMSO‐treated fungi, of which 1,931 DEGs were downregulated and 2,306 DEGs were upregulated (Figure 4b). To identify the biological functions of these DEGs, gene ontology (GO) enrichment analysis was performed. A total of 334 upregulated GO terms and 156 downregulated GO terms were enriched (Table S2). Among the upregulated genes, the GO terms organonitrogen compound metabolic process (GO: 1901564) and organonitrogen compound biosynthetic process (GO: 1901566) were highly enriched (Figure 4c, Table S2). Among the downregulated genes, the GO terms transporter activity (GO: 0005215) and transmembrane transporter activity (GO: 0022857) were the most significantly enriched (Figure 4d, Table S2).

Kyoto Encyclopedia of Genes and Genomes (KEGG) pathway enrichment analysis showed that 55 DEGs, including 47 downregulated and eight upregulated genes, were involved in the “ribosome biogenesis in eukaryotes” KEGG pathway (Figure 4e, Table S3), which was the most enriched pathway among the 96 KEGG pathways detected. Additionally, in the GO category of biological processes, the regulation of ribosome biogenesis, a key signal of cell growth and proliferation, was enriched among the downregulated genes (Figure 4d). Importantly, the genes encoding nucleolar proteins 56 and 58 and U3 small nuclear RNA‐associated proteins were significantly downregulated (Table S3). These ribosomal core proteins combine with small nucleolar RNAs to form small nucleolar ribonucleoproteins that play a crucial role in ribosome biogenesis by guiding the processing and modification of preribosomal RNAs. To further confirm these observations, 10 randomly selected genes involved in ribosome biogenesis were selected for quantitative reverse transcription PCR (RT‐qPCR) analysis (Figure 4f). They were all downregulated, which was consistent with the RNA‐seq data. Additionally, we tested the expression levels of ribosome biogenesis genes in Δ*Fotor2* and Δ*Fofkbp12* lines treated with RAP. RAP had no effect on the expression levels of ribosome biogenesis genes in the Δ*Fofkbp12* mutant, while these genes were downregulated in the Δ*Fotor2* line treated with RAP (Figure S6a). The RNA‐seq results support the previous observations that TOR suppression by RAP inhibits hyphal growth, suggesting that the FoTOR signalling pathway positively regulates ribosome biogenesis and vegetative growth in *F. oxysporum*.

In the process of host infection, *F. oxysporum* secretes a large number of CWDEs to degrade plant cell walls, which facilitates invasion and colonization. The mRNA levels of CWDEs, including cellulases, xylanase, and pectinases, were changed upon TOR inhibition (Table S4). Most of the differentially expressed CWDE genes were downregulated (Table 2). Remarkably, most downregulated CWDE genes encode β‐glucosidases, which are important components of the cellulase system. β‐glucosidase hydrolyses cellobiose by cleaving the β‐(1,4) linkage to generate d‐glucose (Olajuyigbe et al., 2016). In addition, xylanase, endoglucanase, and pectate lyase were dramatically downregulated at the mRNA level (Table 2). The expression levels of CWDE genes displayed a consistent downregulation in the wildtype *F. oxysporum* and Δ*Fotor2* lines treated with RAP. However, RAP had no effect on the expression levels of CWDE genes in the Δ*Fofkbp12* mutant (Figure S6b). These results indicate that FoTOR1 plays an important role in the regulation of CWDEs in *F. oxysporum*.

**TABLE 2:** Representative downregulated differentially expressed genes encoding cell wall‐degrading enzymes, as identified by RNA‐seq

Gene ID	Log_2_(fold change)	Adjusted *p*	Annotation
FOXG_17421	−2.2776	1.21E−4	XYNA_FUSO4 Endo‐1,4‐β‐xylanase A
FOXG_14550	−2.1543	1.69E−21	BGLM_NEOFI Probable β‐glucosidase M
FOXG_09125	−1.8373	5.31E−73	BGLG_ASPOR Probable β‐glucosidase G
FOXG_09571	−1.5896	5.64E−14	BGLF_ASPFC Probable β‐glucosidase F
FOXG_01306	−1.5760	3.85E−35	CRF1_ASPFU Probable glycosidase
FOXG_16582	−1.3076	1.04E−62	AGDC_ASPFU Probable α/β‐glucosidase
FOXG_11081	−1.2542	0.0044	EGLB_ASPOR Probable endo‐β‐1,4‐glucanase B
FOXG_02349	−1.1044	6.70E−30	BGLA_ASPTN Probable β‐glucosidase A
FOXG_04597	−0.9593	0.0384	AGAL2_HYPJE α‐galactosidase 2
FOXG_00531	−0.9080	1.29E−90	GUN_PAEPO Endoglucanase
FOXG_05950	−0.9015	8.12E−147	CRF1_ASPFU Probable glycosidase
FOXG_05948	−0.8788	0.0033	PLYB_ASPOR Probable pectate lyase B
FOXG_15424	−0.8675	1.59E−09	ABFC_ASPTN Probable α‐l‐arabinofuranosidase
FOXG_01365	−0.7566	0.0336	BGLI_NEOFI Probable β‐glucosidase I
FOXG_10604	−0.7525	3.07E−07	BGLI_ASPOR Probable β‐glucosidase I
FOXG_13331	−0.6406	0.0190	PELF2_ASPTN Probable pectin lyase F‐2
FOXG_11735	−0.6363	0.0441	BGALA_NEOFI Probable β‐galactosidase A
FOXG_02278	−0.5686	0.0185	GUB_BACAM β‐glucanase

### 2.5 FoTOR1 interacts with FoTIP4 to regulate ribosome biogenesis in *F. oxysporum*

**FIGURE 5:**
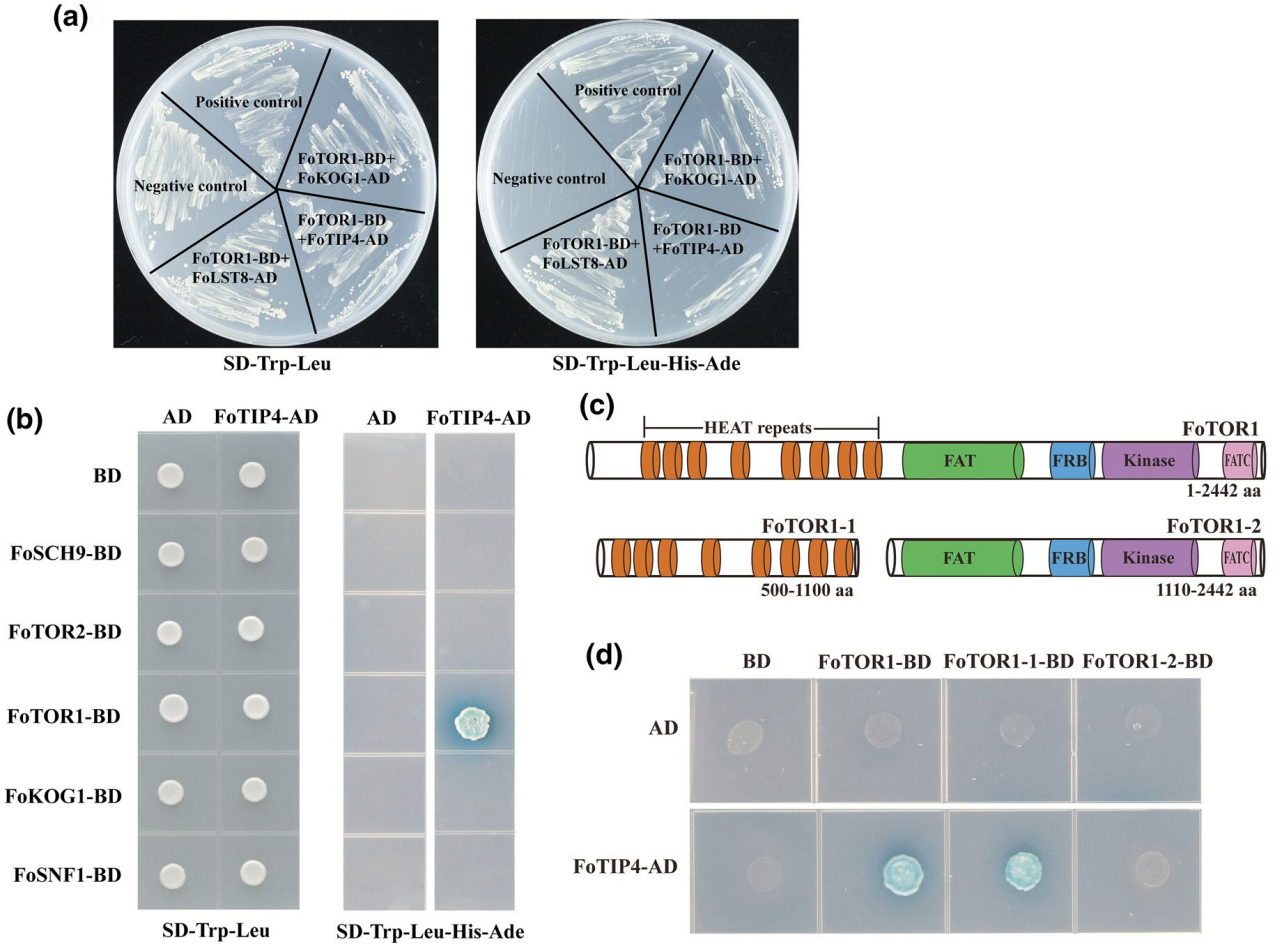
FoTIP4 interacts with FoTOR1 in *Fusarium oxysporum*. (a) Yeast library screening assay. The full‐length FoTOR1 (FOXG_18412) protein was inserted into the BD vector. (b) FoTIP4 interacted with FoTOR1 protein. AD, pGADT7; BD, pGBKT7. BD and AD vectors were used as negative controls. (c) The structures of full‐length FoTOR1 protein (FOXG_18412) and truncated TOR1 protein. FoTOR1‐1 only contains the HEAT repeat domain, and FoTOR1‐2 contains a FAT domain, an FRB domain, a kinase domain, and an FATC domain. (d) FoTIP4 interacted with FoTOR1 and truncated FoTOR1‐1 protein. AD, pGADT7; BD, pGBKT7

To further identify new effectors of the FoTOR signalling pathway, we used the Y2H system to screen for interacting proteins of FoTOR1 in a cDNA library of *F. oxysporum*. The results revealed putative FoTOR1 interacting proteins including FoTIP1/FoKOG1, FoTIP2/FoLST8, and FoTIP3/FoFKBP12. Importantly, a novel FoTOR1 interacting protein 4 (FoTIP4) was detected in *F. oxysporum* (Figure 5a). Sequence alignment revealed that FoTIP4 is homologous to ScSFP1 with low amino acid sequence similarity (18%). SFP1 is a C2H2‐type zinc finger transcription factor that plays an essential role in the control of cell size, regulating the expression of ribosomal proteins and ribosome biogenesis genes in response to nutrient stress, in yeast (Fingerman et al., 2003; Jorgensen et al., 2002; Lempiainen et al., 2009). The gene sequence encoding FoTIP4 (*FOXG_00980*) is located on chromosome 1 in the *F. oxysporum* genome. Further Y2H assays verified the interaction between FoTIP4 and FoTOR1 (Figure 5b). Interestingly, FoTIP4 showed interaction with the HEAT repeat domain of FoTOR1 (Figure 5c,d).

**FIGURE 6:**
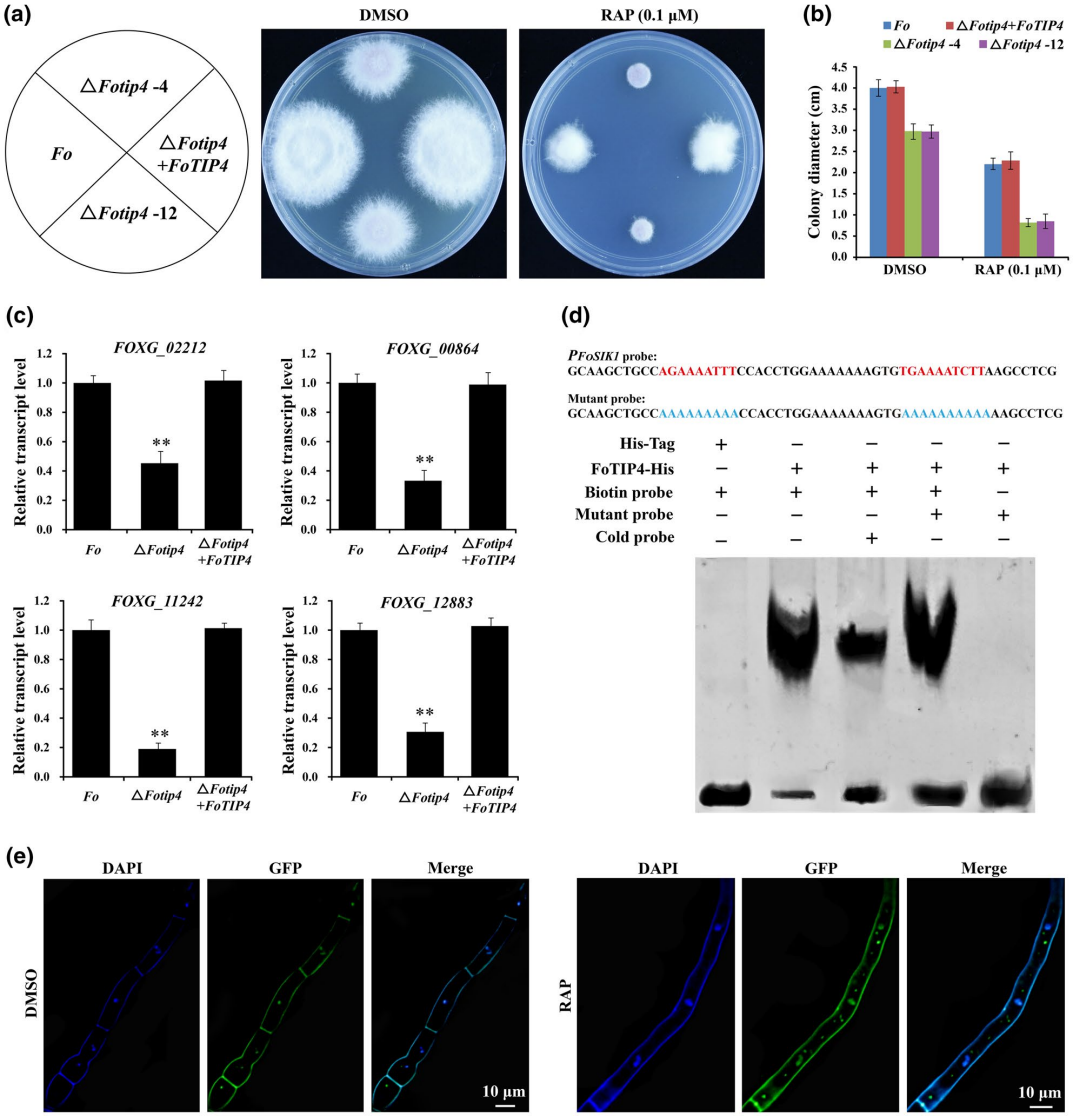
FoTIP4 mediates FoTOR signalling to regulate ribosome biogenesis and hyphal growth in *Fusarium oxysporum*. (a) The growth deficiency and hypersensitivity to RAP of Δ*Fotip4*. The colony morphology of the mutant Δ*Fotip4* strains and the complemented strain upon treatment with RAP or DMSO. (b) Colony diameter of wildtype *F. oxysporum*, Δ*Fotip4* mutants, and the complemented strain were incubated on potato dextrose agar containing DMSO or RAP for 4 days. The data are presented as the mean ± *SD* of *n* = 3 independent experiments. (c) Relative transcript levels of ribosome biogenesis‐related genes in wildtype *F. oxysporum*, Δ*Fotip4* mutants, and the complemented strain. The data are presented as the mean ± *SD* of *n* = 3 independent experiments. **p < 0.01 compared with wildtype *F. oxysporum* (Student’s *t* test). (d) Electrophoretic mobility shift assay showed that FoTIP4 binds to the promoter of *FoSIK1* (*FOXG_12883*) containing the RRPE sequence. The symbols − and + represent absence and presence, respectively. (e) RAP caused FoTIP4 relocalization to the cytoplasm from the nucleus. Hyphae carrying a GFP‐tagged FoTIP4 protein were grown for 3 days in potato dextrose broth, RAP (1 μM) was added, and fungi were incubated for 12 hr. GFP and 4′,6‐diamidino‐2‐phenylindole (DAPI) fluorescence were observed under a confocal laser scanning microscope

To examine the functions of FoTIP4 in *F. oxysporum*, *FoTIP4* deletion mutant strains (Δ*Fotip4*) were generated (Figure S5c,d). Deletion of *FoTIP4* in *F. oxysporum* led to a considerable defect in hyphal growth and a large reduction in biomass on PDA, and the defect was fully restored in the complemented strain (Δ*Fotip4* + *FoTIP4*) (Figure 6a,b). In addition, Δ*Fotip4* strains were more sensitive to RAP and the protein synthesis inhibitor cycloheximide (CHX) than the wildtype *F. oxysporum* strain. Δ*Fotip4* strains displayed a synergistic effect between the *FoTIP4* mutation and RAP treatment, but not between the *FoTIP4* mutation and treatment with the proteasome inhibitor MG‐132. The complemented strain (Δ*Fotip4* + *FoTIP4*) showed no detectable changes in sensitivity to RAP and CHX compared with the wildtype *F. oxysporum* strain (Figures 6a and S7a,b), suggesting that FoTIP4 plays a role in protein synthesis rather than degradation. Furthermore, the number of spores of the Δ*Fotip4* strain was significantly reduced compared with the wildtype *F. oxysporum* strain (Figure S8), implying that FoTIP4 is also involved in the regulation of spore development. The transcript levels of ribosome biogenesis genes were significantly decreased in the Δ*Fotip4* strain compared with the wildtype *F. oxysporum* strain (Figure 6c). ScSFP1 acts as a transcriptional activator of ribosome biogenesis genes whose promoters contain a ribosomal RNA processing element (RRPE) ([A/T]GAAAATTT) and a polymerase A and C (PAC) box (G[C/A]GATGAG) (Fingerman et al., 2003; Jorgensen et al., 2002). Because the promotor of *FoSIK1* (*FOXG_12883*), which is the key regulator of ribosome biogenesis genes, contains the typical RRPE element and was differentially expressed in our RNA‐seq data, we tested whether FoTIP4 can bind to the promoter of *FoSIK1* by electrophoretic mobility shift assay (EMSA). The results showed that FoTIP4 bound to the RRPE element in the promoter of *FoSIK1* (Figure 6d). Other ribosome biogenesis genes containing an RRPE regulatory element, such as *FOXG_00396* and *FOXG_11329*, were downregulated in our transcriptomic data (Table S3), indicating that FoTIP4 positively regulates the expression of ribosome biogenesis genes in *F. oxysporum*. Importantly, reduced FoTOR activity by RAP caused FoTIP4 relocalization to the cytoplasm from the nucleus (Figures 6e and S9), resulting in reduced ribosome biogenesis. These results, combined with the RNA‐seq data, suggest that FoTOR regulates ribosome biogenesis and hyphal growth through interacting with FoTIP4 in *F. oxysporum*.

### 2.6 FoTOR signalling regulates the expression of CWDEs through FoTIP4 in *F. oxysporum*

**FIGURE 7:**
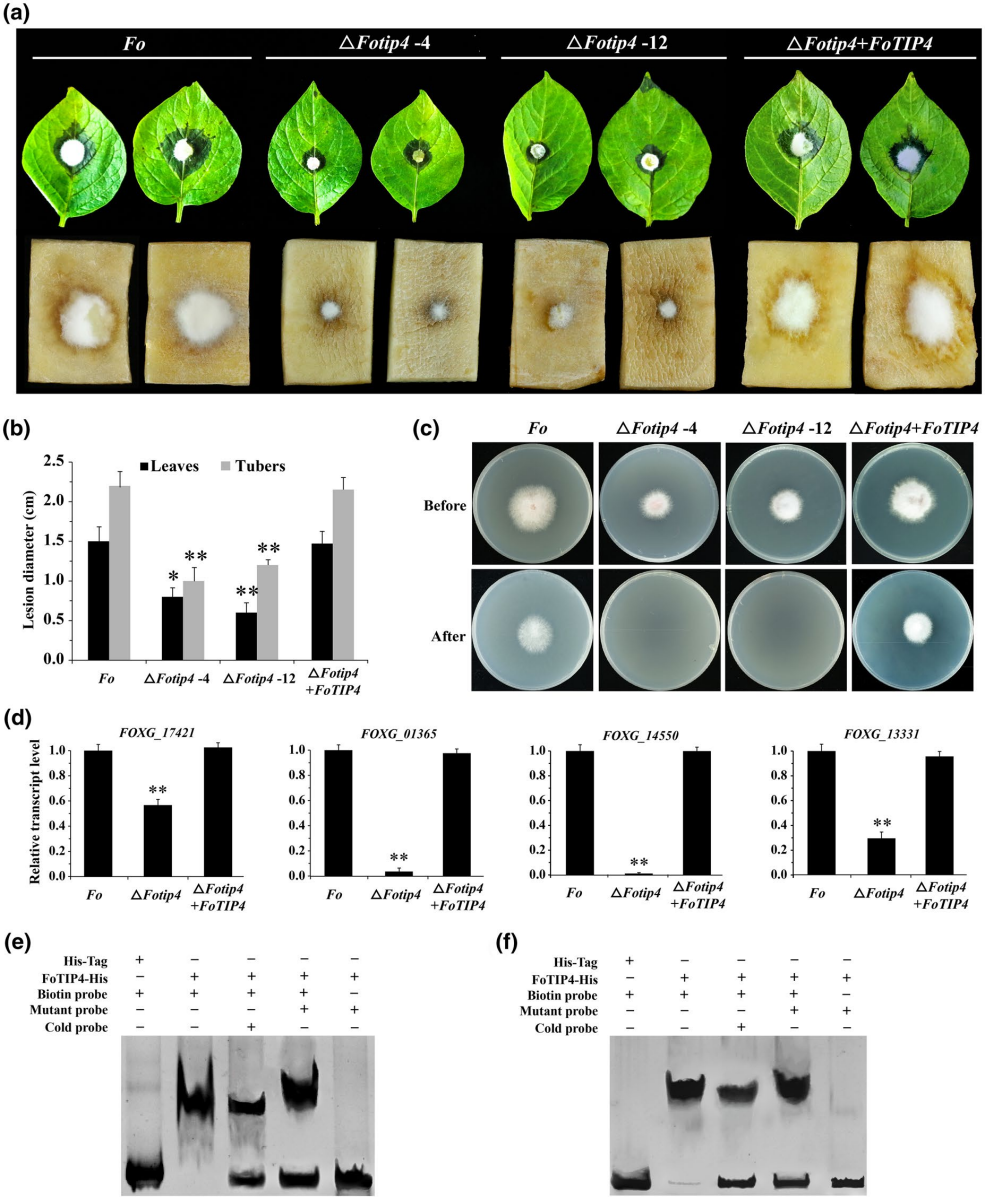
FoTIP4 regulates the pathogenicity of *Fusarium oxysporum*. (a) The pathogenicity of *F. oxysporum* was significantly reduced when FoTIP4 lost its function. Spores of wildtype *F. oxysporum* and Δ*Fotip4* were point‐inoculated on the surface of potato leaves and tubers grown for 4 days. Representative leaves and tubers are shown. Each strain was inoculated on 10 potato leaves or tubers every time, and the experiment was repeated three times. (b) Lesion diameter of potato leaves and tubers. Each column represents the average of 10 potato leaves or tubers. The data are presented as the mean ± *SD* of *n* = 3 independent experiments. (c) Effect of Δ*Fotip4* mutation on penetration of cellophane membranes. Fungal colonies of wildtype *F. oxysporum* and Δ*Fotip4* were grown for 4 days at 28 ℃ on top of cellophane membranes on potato dextrose agar plates (Before), and then the cellophane with the fungal colony was removed and plates were incubated for 2 days to determine the presence of mycelial growth on the plate (After). (d) Relative transcript levels of cell wall‐degrading enzyme (CWDE) genes *FOXG_17421* (endo‐1,4‐β‐xylanase A), *FOXG_01365* (β‐glucosidase I), *FOXG_14550* (β‐glucosidase), and *FOXG_13331* (pectate lyase) in wildtype *F. oxysporum* and Δ*Fotip4* strains. The data are presented as the mean ± *SD* of *n* = 3 independent experiments. **p < 0.01 compared with *F. oxysporum* (Student’s *t* test). (e) The electrophoretic mobility shift assay showed that FoTIP4 binds to the promoter of the β‐glucosidase gene (*FOXG_01365*) containing the PAC box. (f) FoTIP4 binds to the promoter of the pectate lyase gene (*FOXG_13331*) containing the RRPE and PAC boxes. The symbols − and + represent absence and presence, respectively

The FoTOR signalling pathway is a key regulator of the expression of CWDEs in *F. oxysporum*, as shown in Table S4. To determine whether FoTIP4 mediates the effects of the FoTOR signalling pathway in the regulation of CWDEs, we examined the pathogenicity of Δ*Fotip4* mutant strains. In infection assays with potato leaves and tubers, the lesion symptoms of Δ*Fotip4* mutant strains were reduced by approximately 50% compared with those of the wildtype *F. oxysporum* strain (Figure 7a,b). Additionally, a cellophane penetration assay was performed to verify invasive growth of Δ*Fotip4* mutant strains. Efficient penetration was detected in the wildtype and complemented (Δ*Fotip4* + *FoTIP4*) strains, but not in Δ*Fotip4* mutant strains (Figure 7c), suggesting that FoTIP4 plays important roles in the pathogenicity of *F. oxysporum*. Importantly, the main component of cellophane is cellulose, which is a plant cell wall component, implying that FoTIP4 is involved in the regulation of CWDEs in the initial infection process of *F. oxysporum*. We next examined the expression levels of genes encoding CWDEs, including xylanase (*FOXG_17421*), β‐glucosidase (*FOXG_14550* and *FOXG_01365*), and pectin lyase (*FOXG_13331*). These genes were significantly downregulated in the Δ*Fotip4* mutant strain (Figure 7d), confirming the conclusions drawn on the basis of our RNA‐seq data. EMSAs were performed to detect whether FoTIP4 can bind to CWDE promoters containing the RRPE or PAC box. Our results showed that FoTIP4 bound to the RRPE or the PAC box of the *FOXG_01365* and *FOXG_13331* promoters (Figure 7e,f, Table S5). Collectively, these results indicate that FoTOR signalling is involved in the regulation of CWDEs through FoTIP4 in *F. oxysporum*.

## 3 DISCUSSION

TOR is an evolutionarily conserved protein kinase that regulates cell growth and metabolism in response to growth factors, hormones, cellular energy status, and nutrient abundance (Ma & Blenis, 2009; Saxton & Sabatini, 2017). In yeast and animals, TOR is engaged in two large complexes: TORC1 and TORC2. TORC1 mediates temporal control of cell growth by activating anabolic processes such as ribosome biogenesis, protein synthesis, transcription, and translation and by inhibiting catabolic processes such as proteolysis and autophagy (Dowling et al., 2010; Iadevaia et al., 2014; Saxton & Sabatini, 2017). TORC2 controls cell proliferation and survival primarily by phosphorylating several members of the AGC (PKA/PKG/PKC) family of protein kinases. The activation of PKC‐α by TORC2 regulates the cell shape in a cell type‐specific fashion by affecting the actin cytoskeleton (Jacinto et al., 2004). However, little information is known about the TOR signalling pathway in *F. oxysporum*. Based on the recently released genome database of *F. oxysporum*, we identified putative components of the TOR signalling pathway, including TORC1 and TORC2 (Table 1). We found two *TOR* gene homologues (*FOXG_18412* and *FOXG_15946*) in the *F. oxysporum* genome. A previous study showed that high transcript levels of *FoTOR1* (*FOXG_18412*) and very low transcript levels of *FoTOR2* (*FOXG_15946*) were detected after growth in minimal liquid medium containing 25 mM NH_4_NO_3_, and the FoTOR2 protein contains a truncated N‐terminal HEAT repeat domain compared with the FoTOR1 protein (Lopez‐Berges et al., 2010). *FoTOR2* encodes a truncated TOR protein; the FATC domain was not detected in FoTOR2 (Figure 1). Furthermore, deletion of *FoTOR1* may be lethal, while deletion of *FoTOR2* has no effect on hyphal growth in *F. oxysporum*. Phylogenetic analysis showed that FoTOR1 protein was clustered as the core TOR group, while FoTOR2 protein was clustered into the TOR paralogues group; these results were consistent with a previous study (DeIulio et al., 2018). KOG1/RAPTOR functions as a scaffold coupling TOR to substrates in *S. cerevisiae* and animals (González & Hall, 2017). Y2H assays demonstrated that FoTOR1, rather than FoTOR2, interacts with FoKOG1. Interestingly, neither FoTOR1 nor FoTOR2 interacted with FoAVO3 in our Y2H assays, implying that there may be no functional TORC2 in *F. oxysporum*. Additional studies are required to determine the presence of a functional TORC2 complex in *F. oxysporum*. Collectively, the results from the present study indicate that FoTOR1 is a main regulator of the FoTOR signalling pathway in *F. oxysporum*, whereas the function of the FoTOR2 protein remains unclear.

In *S. cerevisiae* and animals, the TOR signalling pathway has been studied in detail (Cornu et al., 2013; De Virgilio & Loewith, 2006; Saxton & Sabatini, 2017; Wullschleger et al., 2006). Several specific drugs have been developed to control TOR activity, such as RAP, Torin1, KU, and AZD (Chresta et al., 2010; Garcia‐Martinez et al., 2009; Heitman et al., 1991a). We applied TOR‐specific inhibitors to detect FoTOR activity in *F. oxysporum*. RAP and Torin1 can effectively inhibit mycelial growth of *F. oxysporum* in a dose‐dependent manner (Figure 2). In this study, we found that the *FoFKBP12* deletion mutant was resistant to RAP (Figure 3). Resistance of *FKBP12* deletion mutants to RAP has been reported in other fungi, including *B. cinerea*, *F. graminearum*, *Fusarium fujikuroi*, and *Mucor circinelloides* (Bastidas et al., 2012; Melendez et al., 2009; Teichert et al., 2006; Yu et al., 2014). Consistent with the *FgFKBP12* deletion mutant of *F*. *graminearum* (Yu et al., 2014), the *FoFKBP12* deletion did not affect mycelial growth and development. Similar to the interaction between FgFKBP12 and FgTOR, RAP is required for the interaction between FoFKBP12 and FoTOR1, but FoFFKBP12 was unable to interact with FoTOR2 with or without RAP, further indicating that FoTOR1 is an essential component of functional TOR complexes in *F. oxysporum*.

RAP can specifically inhibit FoTOR activity at a low concentration in *F. oxysporum*; therefore, RAP was employed to elucidate the function of FoTOR. RNA‐seq analysis showed that FoTOR inhibition results in changes in many metabolic processes (Figure 4), which further indicates that FoTOR plays a key role in growth and development. The ribosome content of a cell potentially imposes an upper limit on the rate of protein synthesis it can sustain. Ribosome biosynthesis and protein synthesis are very energy‐intensive processes; TORC1 regulates ribosome content and protein synthesis by responding to the cellular energy status (Chauvin et al., 2014; Iadevaia et al., 2014). Application of RAP mimics nutrient limitation and inhibited FoTORC1 activity, which decreases the mRNA expression levels of ribosome biogenesis genes (Figure 4). Moreover, CWDEs were significantly downregulated upon FoTOR inhibition, showing that FoTOR plays roles in the regulation of the pathogenicity of *F. oxysporum*. Similarly, TOR inhibition reduces mycelial growth and pathogenicity in other fungi, such as *B. cinerea*, *V. dahliae*, and *F*. *graminearum* (Li et al., 2019; Xiong et al., 2019; Yu et al., 2014).

In this study, we identified a novel FoTOR1 interacting protein, FoTIP4, in a yeast library screening (Figure 5). It is well known that KOG1, as the recruitment protein of TORC1, can recruit substrates to TOR. We found that FoTIP4 interacts with FoTOR1 rather than FoKOG1, implying that FoTIP4 is a new component of FoTORC1. BLASTp analysis showed that FoTIP4 is a unique transcription factor in vascular fungal pathogens. However, there is no homologue of FoTIP4 in the examined mammals, plants, and exogenous fungi such as *Phytophthora infestans*, implying that TIP4 may contribute to mycelial growth and pathogenicity of vascular fungal pathogens. As the homologue of FoTIP4, Sfp1 is phosphorylated by TORC1 and positively regulates the expression of ribosome biogenesis genes in yeast (Albert et al., 2019; Lempiainen et al., 2009). We found that FoTIP4 is a new component of the FoTOR signalling pathway and mediates FoTOR signalling to regulate the expression of ribosome biogenesis and CWDE‐associated genes as well as pathogenicity in *F. oxysporum* (Figures 6 and 7). Consistent with this, we observed that the *FoTIP4* deletion mutant strains (Δ*Fotip4*) displayed considerable defects in hyphal growth and pathogenicity. In addition, EMSAs showed that FoTIP4 can bind to the promoters of ribosome biogenesis and CWDE‐related genes to positively regulate the expression of these genes. In summary, our results provide new insights into FoTOR signalling in the vascular fungus *F. oxysporum*. FoTOR1 may serve as a promising target for controlling and preventing Fusarium wilt caused by *F. oxysporum* in plants.

## 4 EXPERIMENTAL PROCEDURES

### 4.1 Fungal strains and culture conditions

The *F. oxysporum* strain was isolated from Chongqing local potato with dry rot (Fusarium wilt) and was verified through sequencing of the internal transcribed spacer (ITS). The wildtype *F. oxysporum* strain, deletion mutants, and complemented strains were routinely cultured on PDA at 27 °C. For extraction of genomic DNA and conidia production, hyphae were incubated in potato dextrose broth (PDB) at 27 °C with shaking at 160 rpm (Lopez‐Berges et al., 2010).

### 4.2 Construction of vectors for gene deletion and complementation

The primers used to amplify the flanking sequences or coding sequence of each gene are listed in Table S6. Gene deletion and complementation of *F. oxysporum* were carried out as described previously (Luo et al., 2016). *Agrobacterium tumefaciens* AGL‐1 was used to transform the conidia of *F. oxysporum*. *A*. *tumefaciens*‐mediated transformation was performed as described previously (Maruthachalam et al., 2011). Randomly selected transformants were transferred to fresh PDA with hygromycin.

### 4.3 Construction and screening of a Y2H library

Total RNAs were extracted from mycelium of *F. oxysporum* using the RNAprep Kit (TIANGEN). The first‐strand cDNA was obtained using 1–2 μg of total RNA as template. This first‐strand cDNA was then used as template for low‐cycle (c.20) long‐distance PCR (LD‐PCR) amplification to generate 3–6 μg double‐stranded cDNA (dscDNA). These dscDNAs were purified using CHROMA SPIN TE‐400 columns (Takara) to obtain DNA for library construction. The cDNA library for Y2H screening was fused with the GAL4 activation domain of the pGADT7 vector as prey using the Matchmaker Gold Yeast Two‐Hybrid System (Clontech). The *FoTOR1* (*FOXG_18412*) gene was fused with the GAL4 DNA‐binding domain in pGBKT7 to ensure that there was no autoactivation and toxicity, and the FoTOR1 fusion protein was used as bait to identify interacting proteins. Co‐transformation of the library cDNA (prey) and the plasmid pGBKT7‐FoTOR1 (bait) into Y2HGold yeast strain allowed interaction between prey and bait.

### 4.4 Yeast two‐hybrid assay

In order to construct plasmids for Y2H analyses, the coding sequences of genes were amplified from the cDNA of *F. oxysporum*. The genes were inserted into the yeast GAL4 binding domain vector pGBKT7 or the GAL4 activation domain vector pGADT7 (Clontech). Pairs of Y2H plasmids were co‐transformed into *S. cerevisiae* Y2HGold following the PEG/LiAc transformation protocol (Clontech). Transformants were grown at 28 °C for 5 days on synthetic medium lacking leucine and tryptophan (SD−Leu−Trp). Then yeast colonies were transferred to synthetic medium lacking histidine, leucine, tryptophan, and adenine (SD−His−Leu−Trp−Ade) containing 40 µg/ml X‐α‐Gal and 200 ng/ml aureobasidin A as described in the manual (Clontech). The pair of plasmids pGBKT7‐53 and pGADT7‐T was used as a positive control. The pair of plasmids pGBKT7‐Lam and pGADT7‐T was used as a negative control. Three independent experiments were performed.

### 4.5 Fluorescence microscopy

The full‐length *FoTIP4* encoding sequence was subcloned downstream of the *FoTIP4* promoter in Gateway entry vector p8GWN to generate *PFoTIP4::FoTIP4‐eGFP*. These recombinant constructs were transformed into pEarleyGate303 through LR recombination reactions (Ren et al., 2011). The plasmids were introduced into *A*. *tumefaciens* AGL‐1 and the conidia of *F. oxysporum* were transformed as described previously. Transformants were transferred to PDA containing hygromycin for further analysis. Green fluorescent protein (GFP) and 4′,6‐diamidino‐2‐phenylindole (DAPI) fluorescence were observed using a confocal laser scanning microscope (Olympus; Fluoview FV1200).

### 4.6 Electrophoretic mobility shift assay

The full‐length *FoTIP4* coding sequence was inserted in the vector pCold TF (Takara) and expressed in *Escherichia coli* BL21 (DE3) by incubation with 0.5 mM IPTG for 5 hr at 16 °C. The recombinant protein was purified with a His60 Ni Gravity Column (Takara) according to the manufacturer’s instructions. The probes containing an RRPE or PAC box derived from *FoSIK1* (*FOXG_12883*), pectate lyase gene (*FOXG_13331*), and β‐glucosidase gene (*FOXG_01365*) promoters were labelled with biotin using the EMSA Probe Biotin Labeling Kit (Beyotime). The same unlabelled DNA fragment was used as a competitor, while the RRPE or PAC box within a probe changed into AAAAAAAA was used as a negative control. The EMSA was performed using the electrophoretic mobility shift assay kit (Beyotime) according to the manufacturer’s instructions.

### 4.7 Transcriptome sequencing and analysis

Hyphae of *F. oxysporum* were grown for 4 days in PDB at 27 °C with shaking at 160 rpm, treated with 1 µM RAP or DMSO (as a control), and incubated for 12 hr. Total RNA of *F. oxysporum* mycelium was isolated using the RNAprep Pure Plant Kit (TIANGEN). For each treatment, three independent biological replicates were performed. An Illumina HiSeq 2000 platform was used to sequence the cDNA library, and 100‐bp paired‐end reads were generated. The clean reads were mapped to the *F. oxysporum* reference genome using TopHat2 software. Cufflinks and Cuffdiff were used to assemble the mapped reads and identify DEGs, respectively. GO enrichment (corrected *p* value < 0.05) of the DEGs was performed using GOseq software. The KEGG pathway enrichment of DEGs (corrected *p* value < 0.05) was obtained using KOBAS software (Mao et al., 2005).

### 4.8 RT‐qPCR

Total RNA of *F. oxysporum* mycelium treated for 12 hr in PDB containing DMSO or RAP (1 μM) was isolated using the RNAprep Pure Plant Kit (TIANGEN). Relative transcript levels were assayed by one‐step real‐time PCR analysis using the CFX96 real‐time PCR system (Bio‐Rad). Real‐time PCR primers were designed using Primer Premier v. 5.0 (details are presented in Table S6). *FoEIF1α* was used as an internal control. The data are presented as the mean ± *SD* of three independent experiments.

### 4.9 Combination index value measurement

CI values were used to quantitatively measure the interaction between Torin1 and RAP. The interaction is categorized as synergism (CI < 1), additive effects (CI = 1), or antagonism (CI > 1) (Chou, 2006). Hyphae of *F. oxysporum* were incubated on PDA containing different concentrations of Torin1 or RAP or a combination of Torin1 and RAP for 6 days at 27 °C. Colony diameter was measured to calculate growth inhibition. Experiments were repeated three times. Fa represents the fraction of colony diameter affected by the reagent. The Fa values, which were calculated using CompuSyn software, indicate growth inhibition.

### 4.10 Pathogen inoculation and cellophane invasion assays

Pathogen inoculation was performed by point inoculation on the surface of potato leaves and tubers with conidia of wildtype *F. oxysporum*, Δ*Fotip4* mutants, and the complemented strain (Δ*Fotip4* + *FoTIP4*) (10^7^ conidia/ml) as described previously (Thatcher et al., 2009). Inoculated leaves and tubers were cultured on moist filter paper at 27 °C in a short daylight condition for 4 days. Each strain was inoculated on at least 10 potato leaves or tubers every time. The cellophane invasion assay was performed as described previously (Prados Rosales & Di, 2008). Each experiment was repeated at least three times.

## AUTHOR CONTRIBUTIONS

M.R. and L.L. designed the experiments. L.L., T.Z., and Y.S. performed the experiments. L.L., T.Z., Y.S., and X.L. analysed the data. L.L., T.Z., R.D., and M.R. wrote the manuscript.

## Supporting information

**FIGURE S1** A phylogenetic tree of TOR proteins constructed by the neighbour‐joining (NJ) algorithm using TOR kinase domain sequence alignment with 1,000 bootstrap replicates. The TOR protein kinase domain sequences of 14 *Fusarium oxysporum* isolates, *Schizosaccharomyces pombe*, *Saccharomyces cerevisiae*, *Verticillium dahliae*, *Fusarium graminearum*, and *Homo sapiens* were used to perform phylogenetic analysis. The core and lineage‐specific (LS) clades of TOR kinase in *F. oxysporum* are shown in boxes

**FIGURE S2** Yeast two‐hybrid analysis of the interaction between FoTORs and FoKOG1 or FoAVO3. Yeast colonies transferred with the bait and prey constructs were assayed for growth on yeast SD−His−Leu−Trp−Ade medium containing 40 µg/ml X‐α‐Gal and 200 ng/ml aureobasidin A at 28 °C for 3 days. AD, pGADT7; BD, pGBKT7

**FIGURE S3** TOR inhibitors RAP and Torin1 inhibited germination and production of spores. (a) and (b) Germination rate and number of spores of *Fusarium oxysporum* treated with RAP (0.1 μM) and Torin1 (20 μM) for 48 hr. Representative photos are shown. The data are presented as the mean ± *SD* of *n* = 3 independent experiments. (c) Relative transcript levels of sporulation‐related genes of *F. oxysporum* treated with RAP (0.1 μM) and Torin1 (20 μM) for 12 hr. The data are presented as the mean ± *SD* of *n* = 3 independent experiments. *P < 0.05, **P < 0.01 compared with the DMSO group (Student’s *t*‐test)

**FIGURE S4** The growth trends of the Δ*Fotor2* mutants were the same as that of the wild‐type *Fusarium oxysporum* strain upon RAP and Torin1 treatment. (a) The phenotypes of the wild‐type *F. oxysporum*, Δ*Fotor2*, and complemented (Com) strains. Conidia of *F. oxysporum* were incubated on potato dextrose agar (PDA) containing RAP and Torin1 for 6 days. (b) Colony diameter of *F. oxysporum* incubated on PDA with RAP and Torin1 for 6 days. The data are presented as the mean ± *SD* of *n* = 3 independent experiments

**FIGURE S5** Verification of Δ*Fofkbp12* and Δ*Fotip4*. (a) Gel electrophoresis of the *FKBP12* gene and the hygromycin resistance (*Hyg*) cassette. The *FoFKBP12* gene and the *Hyg* cassette were amplified from wild‐type *Fusarium oxysporum*, Δ*fkbp12* mutants, and complemented (Com) strains with *FKBP12* F/R and *Hyg* F/R primers, respectively. M, DNA marker; *Fo*, *F. oxysporum*; Com, complemented. (b) Quantitative reverse transcription PCR (RT‐qPCR) analysis of *FKBP12* gene expression in wild‐type *F. oxysporum*, Δ*fkbp12* mutants, and Com strains. The data are presented as the mean ± *SD* of *n* = 3 independent experiments. (c) Gel electrophoresis of the *TIP4* gene and the *Hyg* cassette. The *FoTIP4* gene and the *Hyg* cassette were amplified from wild‐type *F. oxysporum*, Δ*tip4* mutants, and Com strains with *TIP4* F/R and *Hyg* F/R primers, respectively. M, DNA marker; Com, complemented. (d) RT‐qPCR analysis of *TIP4* gene expression in wild‐type *F. oxysporum*, Δ*tip4* mutants, and Com strains. The data are presented as the mean ± *SD* of *n* = 3 independent experiments

**FIGURE S6** The relative transcript levels of ribosome biogenesis and cell wall‐degrading enzyme (CWDE) genes in Δ*Fotor2* and Δ*Fofkbp12* lines. (a) The relative transcript levels of the ribosome biogenesis genes *FOXG_11242* and *FOXG_12883* in Δ*Fotor2* and Δ*Fofkbp12* lines. (b) The relative transcript levels of the CWDE genes *FOXG_01365* and *FOXG_17421* in Δ*Fotor2* and Δ*Fofkbp12* lines. The data are presented as the mean ± *SD* of *n* = 3 independent experiments

**FIGURE S7** FoTIP4 is involved in protein biosynthesis. (a) The Δ*Fotip4* strain was more sensitive to the protein synthesis inhibitor cycloheximide (CHX), but not to the proteasome inhibitor MG‐132, than the wild‐type strain. Hyphae of wild‐type *Fusarium oxysporum* and Δ*Fotip4* were incubated on potato dextrose agar (PDA) containing DMSO, CHX, or MG‐132 for 6 days. (b) Colony diameter of wild‐type *F. oxysporum* and Δ*Fotip4* were incubated on PDA containing DMSO, CHX (100 μM), or MG‐132 (50 μM) for 6 days. The data are presented as the mean ± *SD* of *n* = 3 independent experiments

**FIGURE S8** Number of spores of wild‐type *Fusarium oxysporum*, Δ*Fotip4* mutants, and the complemented strain (Δ*Fotip4* + *FoTIP4*) for 5 days. The data are presented as the mean ± *SD* of *n* = 3 independent experiments. **P < 0.01 compared with wild‐type *F. oxysporum* (Student’s *t*‐test)

**FIGURE S9** Quantification of FoTIP4‐GFP fluorescence in the nucleus and cytoplasm. Hyphae carrying a GFP‐tagged FoTIP4 were grown for 3 days in potato dextrose broth, RAP (1 μM) was added, and fungi were incubated for 12 hr. Data are presented as the mean ± *SD* of *n* = 2 independent experiments. **P < 0.01 compared with the DMSO group (Student’s *t*‐test)

**TABLE S1***TOR* genes in formae speciales of *Fusarium oxysporum*

**TABLE S2** Enriched GO terms among differentially expressed genes

**TABLE S3** Differentially expressed genes involved in ribosome biogenesis in eukaryotes

**TABLE S4** Differentially expressed genes encoding cellulase, hemicellulose, and pectinase, as identified by RNA‐seq

**TABLE S5** Probe sequences of *FOXG_01365* and *FOXG_13331* promoters

**TABLE S6** Primers used for cloning and reverse transcription PCR in this study

## Data Availability

The data that support the findings of this study are available from the corresponding author upon reasonable request.
