# Supplementary material for: Target of rapamycin controls hyphal growth and pathogenicity through FoTIP4 in Fusarium oxysporum
Source: Mol Plant Pathol. 2021 Jul 20;22(10):1239–55. doi: 10.1111/mpp.13108 (PMC8435236; doi:10.1111/mpp.13108)
Supplement: Supplementary file 1 — FIGURE S1 A phylogenetic tree of TOR proteins constructed by the neighbour‐joining (NJ) algorithm using TOR kinase domain sequence alignment with 1,000 bootstrap replicates. The TOR protein kinase domain sequences of 14 Fusarium oxysporum isolates, Schizosaccharomyces pombe, Saccharomyces cerevisiae, Verticillium dahliae, Fusarium graminearum, and Homo sapiens were used to perform phylogenetic analysis. The core and lineage‐specific (LS) clades of TOR kinase in F. oxysporum are shown in boxes [file MPP-22-1239-s013.docx]

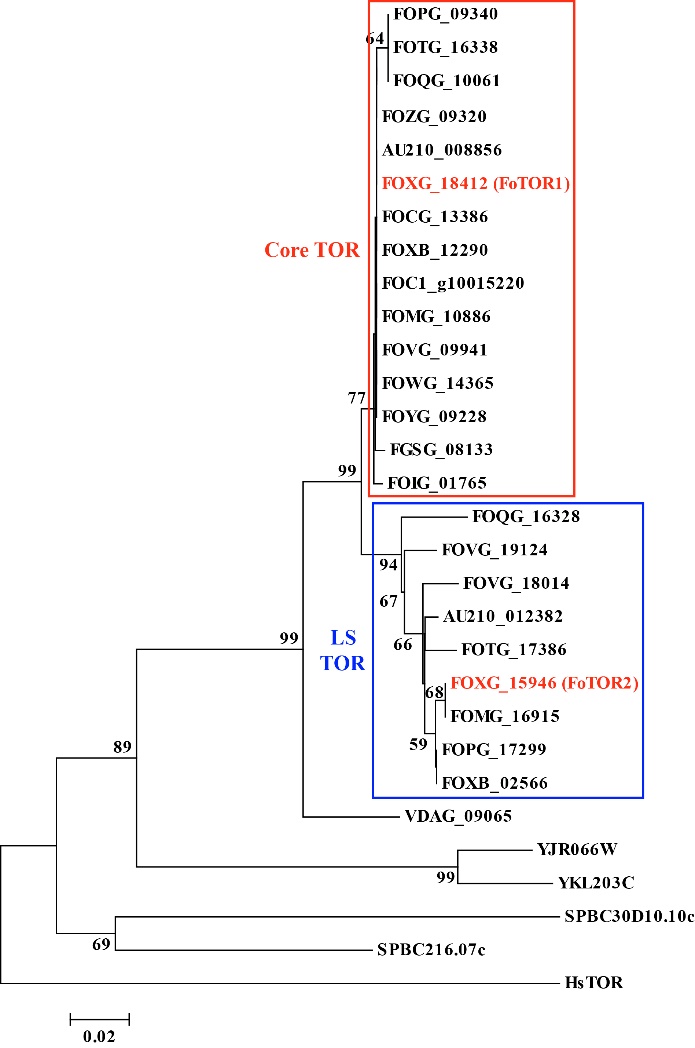


**Figure S1 A phylogenetic tree of TOR proteins constructed by the neighbor-joining (NJ) algorithm using TOR kinase domain sequence alignment, 1000 of bootstrap replicates.** The TOR protein kinase domain sequences of 14 *Fusarium oxysporum* isolates, *Schizosaccharomyces pombe*, *Saccharomyces cerevisiae*, *Verticillium dahliae*, *Fusarium graminearum* and *Homo sapiens* were used to perform phylogenetic analysis. The core and lineage-specific (LS) clades of TOR kinase in *Fusarium oxysporum* are shown in boxes.
