# Supplementary material for: Target of rapamycin controls hyphal growth and pathogenicity through FoTIP4 in Fusarium oxysporum
Source: Mol Plant Pathol. 2021 Jul 20;22(10):1239–55. doi: 10.1111/mpp.13108 (PMC8435236; doi:10.1111/mpp.13108)
Supplement: Supplementary file 2 — FIGURE S2 Yeast two‐hybrid analysis of the interaction between FoTORs and FoKOG1 or FoAVO3. Yeast colonies transferred with the bait and prey constructs were assayed for growth on yeast SD−His−Leu−Trp−Ade medium containing 40 µg/ml X‐α‐Gal and 200 ng/ml aureobasidin A at 28 °C for 3 days. AD, pGADT7; BD, pGBKT7 [file MPP-22-1239-s004.docx]

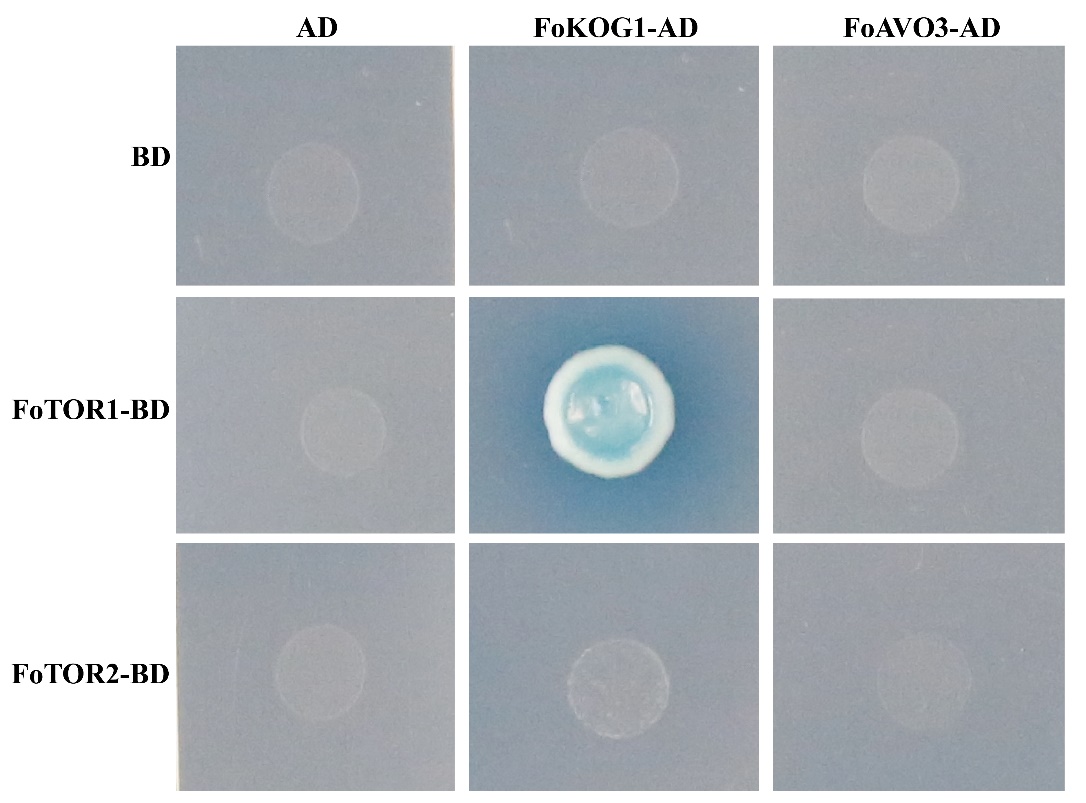


**Figure S2** **Yeast two-hybrid analysis of the interaction between FoTORs and FoKOG1 or FoAVO3.** Yeast colonies transferred with the bait and prey constructs were assayed for growth on yeast SD/-His-Leu-Trp-Ade medium containing 40 µg/ml X-a-Gal and 200 ng/ml Aureobasidin at 28 °C for 3 days. AD: pGADT7, BD: pGBKT7.
