# Supplementary material for: Target of rapamycin controls hyphal growth and pathogenicity through FoTIP4 in Fusarium oxysporum
Source: Mol Plant Pathol. 2021 Jul 20;22(10):1239–55. doi: 10.1111/mpp.13108 (PMC8435236; doi:10.1111/mpp.13108)
Supplement: Supplementary file 3 — FIGURE S3 TOR inhibitors RAP and Torin1 inhibited germination and production of spores. (a) and (b) Germination rate and number of spores of Fusarium oxysporum treated with RAP (0.1 μM) and Torin1 (20 μM) for 48 hr. Representative photos are shown. The data are presented as the mean ± SD of n = 3 independent experiments. (c) Relative transcript levels of sporulation‐related genes of F. oxysporum treated with RAP (0.1 μM) and Torin1 (20 μM) for 12 hr. The data are presented as the mean ± SD of n = 3 independent experiments. *P < 0.05, **P < 0.01 compared with the DMSO group (Student’s t‐test) [file MPP-22-1239-s009.docx]

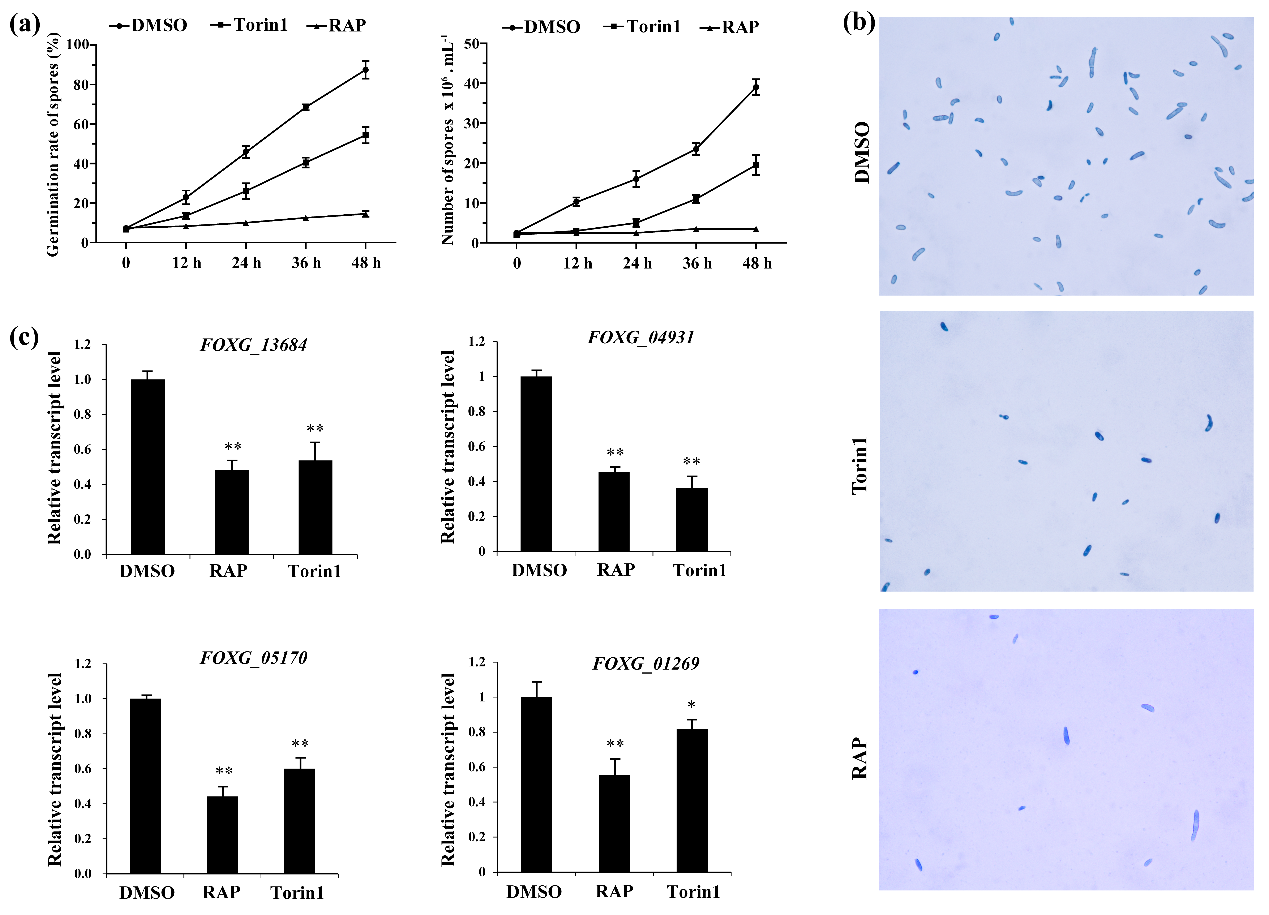


**Figure S3 TOR inhibitors RAP and Torin1 inhibited germination and production of spores.** **(a)** and **(b)** Germination rate and number of spores of *F. oxysporum* treated with RAP (0.1 μM) and Torin1 (20 μM) for 48 h. Representative photos were shown. The data represent the mean ± SD of n = 3 independent experiments. **(c)** Relative transcript level of sporulation related genes of *F. oxysporum* treated with RAP (0.1 μM) and Torin1 (20 μM) for 12 h. The data represent the mean ± SD of n = 3 independent experiments. Asterisks denote student’s *t* test signiﬁcant difference compared with DMSO (*P < 0.05; **P < 0.01).
