# Supplementary material for: Target of rapamycin controls hyphal growth and pathogenicity through FoTIP4 in Fusarium oxysporum
Source: Mol Plant Pathol. 2021 Jul 20;22(10):1239–55. doi: 10.1111/mpp.13108 (PMC8435236; doi:10.1111/mpp.13108)
Supplement: Supplementary file 4 — FIGURE S4 The growth trends of the ΔFotor2 mutants were the same as that of the wild‐type Fusarium oxysporum strain upon RAP and Torin1 treatment. (a) The phenotypes of the wild‐type F. oxysporum, ΔFotor2, and complemented (Com) strains. Conidia of F. oxysporum were incubated on potato dextrose agar (PDA) containing RAP and Torin1 for 6 days. (b) Colony diameter of F. oxysporum incubated on PDA with RAP and Torin1 for 6 days. The data are presented as the mean ± SD of n = 3 independent experiments [file MPP-22-1239-s006.docx]

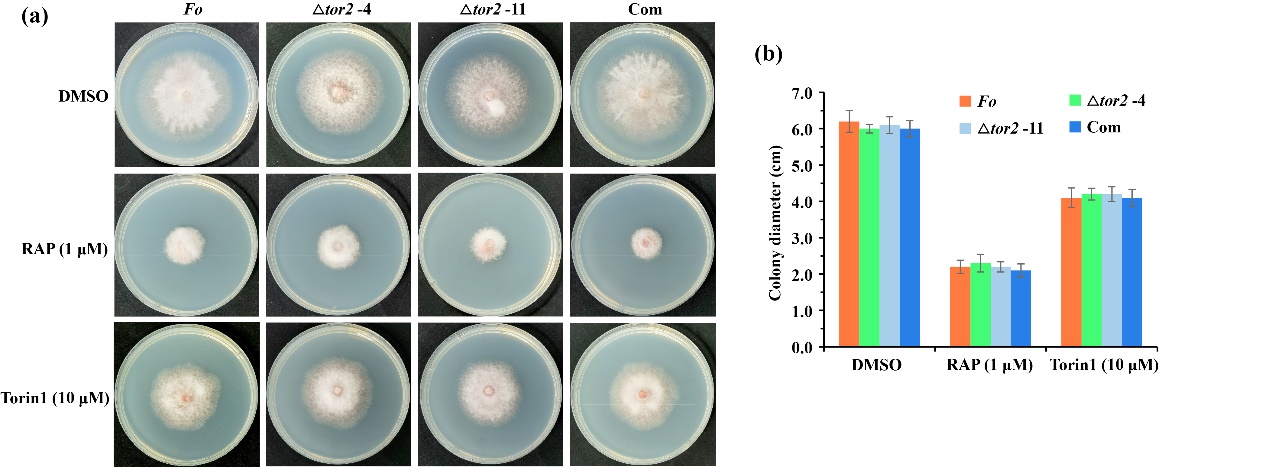


**Figure S4 The growth trends of the Δ*Fotor2* mutants was the same as that of the *Fo* strain in RAP and Torin1 treatment.** **(a)** The phenotypes of the *Fo*, Δ*Fotor2* and complementary strains (Com). Conidia of *F. oxysporum* were incubated on PDA medium containing RAP and Torin1 for 6 days. **(b)** Colony diameter of *F. oxysporum* was incubated on PDA medium with RAP and Torin1 for 6 days. The data represent the mean ± SD of n = 3 independent experiments.
