# Supplementary material for: Target of rapamycin controls hyphal growth and pathogenicity through FoTIP4 in Fusarium oxysporum
Source: Mol Plant Pathol. 2021 Jul 20;22(10):1239–55. doi: 10.1111/mpp.13108 (PMC8435236; doi:10.1111/mpp.13108)
Supplement: Supplementary file 5 — FIGURE S5 Verification of ΔFofkbp12 and ΔFotip4. (a) Gel electrophoresis of the FKBP12 gene and the hygromycin resistance (Hyg) cassette. The FoFKBP12 gene and the Hyg cassette were amplified from wild‐type Fusarium oxysporum, Δfkbp12 mutants, and complemented (Com) strains with FKBP12 F/R and Hyg F/R primers, respectively. M, DNA marker; Fo, F. oxysporum; Com, complemented. (b) Quantitative reverse transcription PCR (RT‐qPCR) analysis of FKBP12 gene expression in wild‐type F. oxysporum, Δfkbp12 mutants, and Com strains. The data are presented as the mean ± SD of n = 3 independent experiments. (c) Gel electrophoresis of the TIP4 gene and the Hyg cassette. The FoTIP4 gene and the Hyg cassette were amplified from wild‐type F. oxysporum, Δtip4 mutants, and Com strains with TIP4 F/R and Hyg F/R primers, respectively. M, DNA marker; Com, complemented. (d) RT‐qPCR analysis of TIP4 gene expression in wild‐type F. oxysporum, Δtip4 mutants, and Com strains. The data are presented as the mean ± SD of n = 3 independent experiments [file MPP-22-1239-s002.docx]

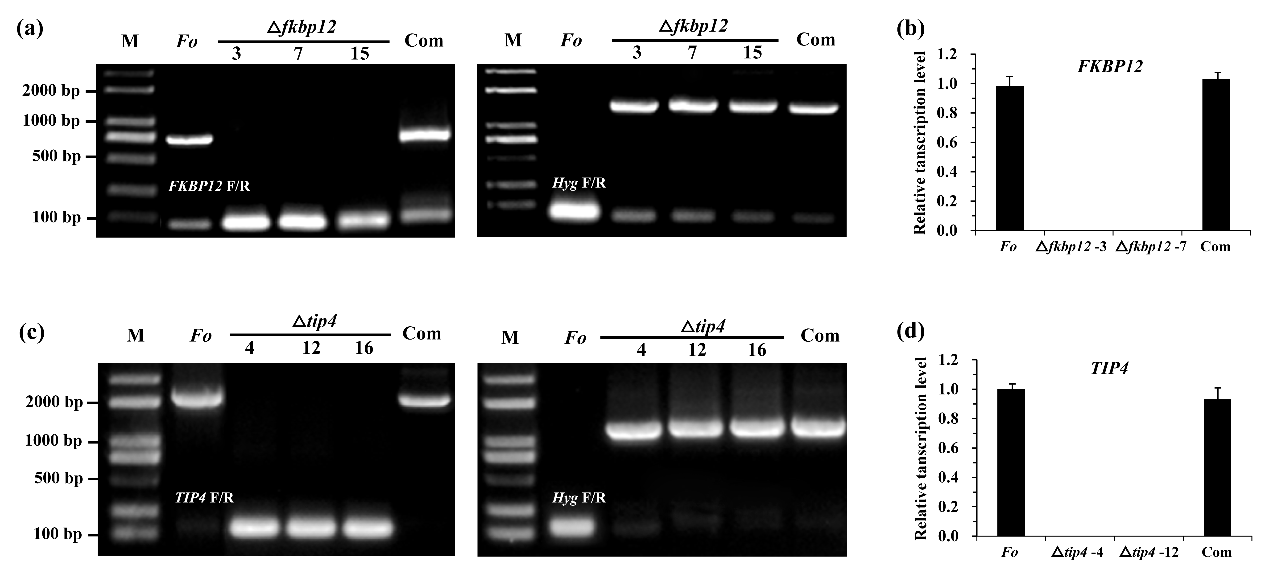


**Figure S5 Verification of Δ*Fofkbp12* and Δ*Fotip4*.** **(a)** Gel electrophoresis of *FKBP12* gene and *Hyg* cassette. *FoFKBP12* gene and *Hyg* cassette were amplified from *Fo*, Δ*fkbp12* mutants and Com with *FKBP12* F/R and *Hyg* F/R primers, individually. M, DNA marker, *Fo*, *F. oxysporum*; Com, complementary strain. **(b)** qRT-PCR analysis of *FKBP12* gene expression in the *Fo*, Δ*fkbp12* mutants and Com. The data represent the mean ± SD of n = 3 independent experiments. **(c)** Gel electrophoresis of *TIP4* gene and *Hyg* cassette. *FoTIP4* gene and *Hyg* cassette were amplified from *Fo*, Δ*tip4* mutants and Com with *TIP4* F/R and *Hyg* F/R primers, individually. M, DNA marker, *Fo*, *F. oxysporum*; Com, complementary strain. **(d)** qRT-PCR analysis of *TIP4* gene expression in the *Fo*, Δ*tip4* mutants and Com. The data represent the mean ± SD of n = 3 independent experiments.
