# Supplementary material for: Target of rapamycin controls hyphal growth and pathogenicity through FoTIP4 in Fusarium oxysporum
Source: Mol Plant Pathol. 2021 Jul 20;22(10):1239–55. doi: 10.1111/mpp.13108 (PMC8435236; doi:10.1111/mpp.13108)
Supplement: Supplementary file 6 — FIGURE S6 The relative transcript levels of ribosome biogenesis and cell wall‐degrading enzyme (CWDE) genes in ΔFotor2 and ΔFofkbp12 lines. (a) The relative transcript levels of the ribosome biogenesis genes FOXG_11242 and FOXG_12883 in ΔFotor2 and ΔFofkbp12 lines. (b) The relative transcript levels of the CWDE genes FOXG_01365 and FOXG_17421 in ΔFotor2 and ΔFofkbp12 lines. The data are presented as the mean ± SD of n = 3 independent experiments [file MPP-22-1239-s012.docx]

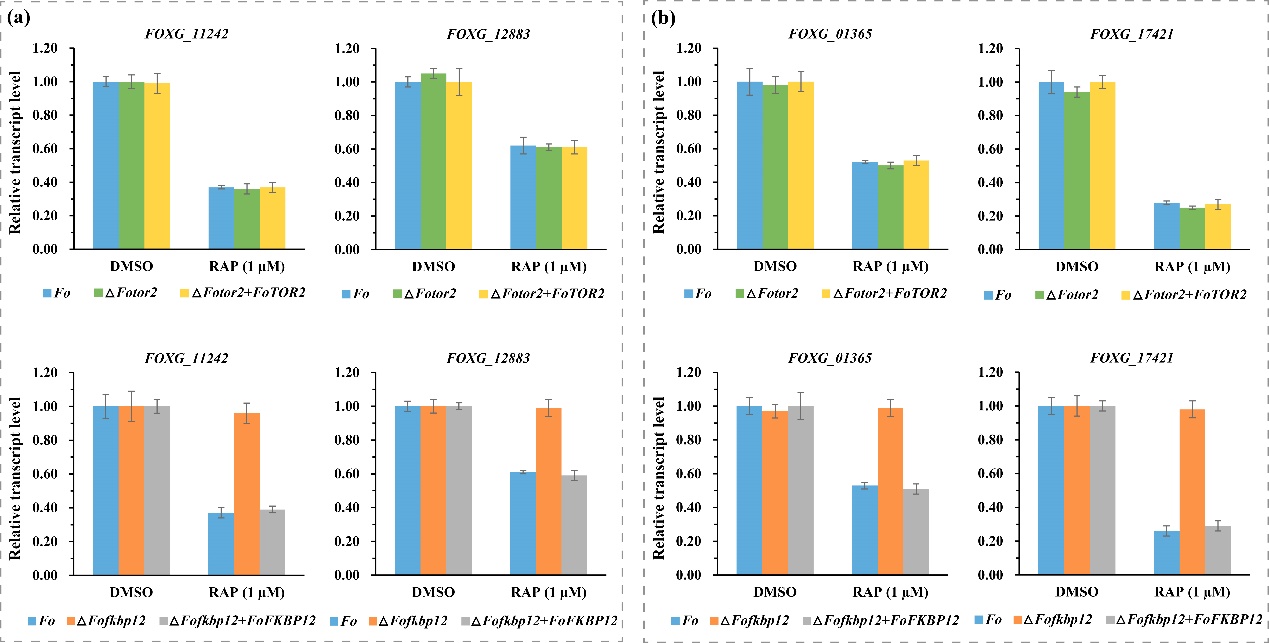


**Figure S6 The relative transcript levels of ribosome biogenesis and CWDE genes in Δ*Fotor2* and Δ*Fofkbp12* lines. (a)** The relative transcript levels of ribosome biogenesis *FOXG_11242* and *FOXG_12883* genes in Δ*Fotor2* and Δ*Fofkbp12* lines. **(b)** The relative transcript levels of CWDE *FOXG_01365* and *FOXG_17421* genes in Δ*Fotor2* and Δ*Fofkbp12* lines. The data represent the mean ± SD of n = 3 independent experiments.
