# Supplementary material for: Target of rapamycin controls hyphal growth and pathogenicity through FoTIP4 in Fusarium oxysporum
Source: Mol Plant Pathol. 2021 Jul 20;22(10):1239–55. doi: 10.1111/mpp.13108 (PMC8435236; doi:10.1111/mpp.13108)
Supplement: Supplementary file 7 — FIGURE S7 FoTIP4 is involved in protein biosynthesis. (a) The ΔFotip4 strain was more sensitive to the protein synthesis inhibitor cycloheximide (CHX), but not to the proteasome inhibitor MG‐132, than the wild‐type strain. Hyphae of wild‐type Fusarium oxysporum and ΔFotip4 were incubated on potato dextrose agar (PDA) containing DMSO, CHX, or MG‐132 for 6 days. (b) Colony diameter of wild‐type F. oxysporum and ΔFotip4 were incubated on PDA containing DMSO, CHX (100 μM), or MG‐132 (50 μM) for 6 days. The data are presented as the mean ± SD of n = 3 independent experiments [file MPP-22-1239-s015.docx]

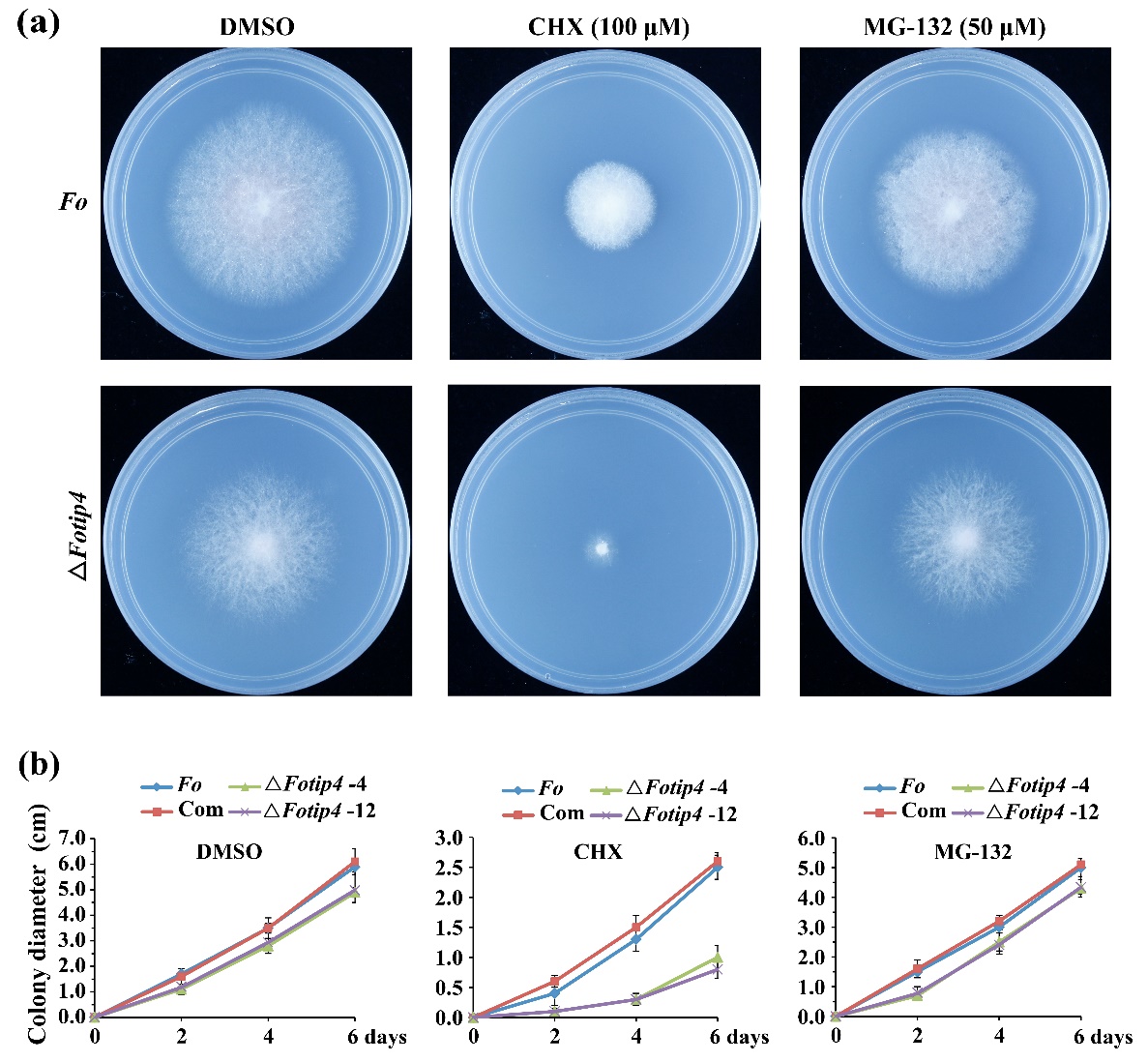


**Figure S7** **FoTIP4 involved in the biosynthesis of protein. (a)** Δ*Fotip4* strain was more sensitive to the protein synthesis inhibitor cycloheximide (CHX) than wild type strain, but not to the proteasome inhibitor MG-132. Hyphae of *Fo* and Δ*Fotip4* were incubated on PDA including DMSO, CHX and MG-132 for 6 days. (**b)** Colony diameter of *Fo* and Δ*Fotip4* were incubated on PDA medium including DMSO, CHX (100 μM) and MG-132 (50 μM) for 6 days. The data represent the mean ± SD of n = 3 independent experiments.
