# Supplementary material for: Target of rapamycin controls hyphal growth and pathogenicity through FoTIP4 in Fusarium oxysporum
Source: Mol Plant Pathol. 2021 Jul 20;22(10):1239–55. doi: 10.1111/mpp.13108 (PMC8435236; doi:10.1111/mpp.13108)
Supplement: Supplementary file 8 — FIGURE S8 Number of spores of wild‐type Fusarium oxysporum, ΔFotip4 mutants, and the complemented strain (ΔFotip4 + FoTIP4) for 5 days. The data are presented as the mean ± SD of n = 3 independent experiments. **P < 0.01 compared with wild‐type F. oxysporum (Student’s t‐test) [file MPP-22-1239-s007.docx]

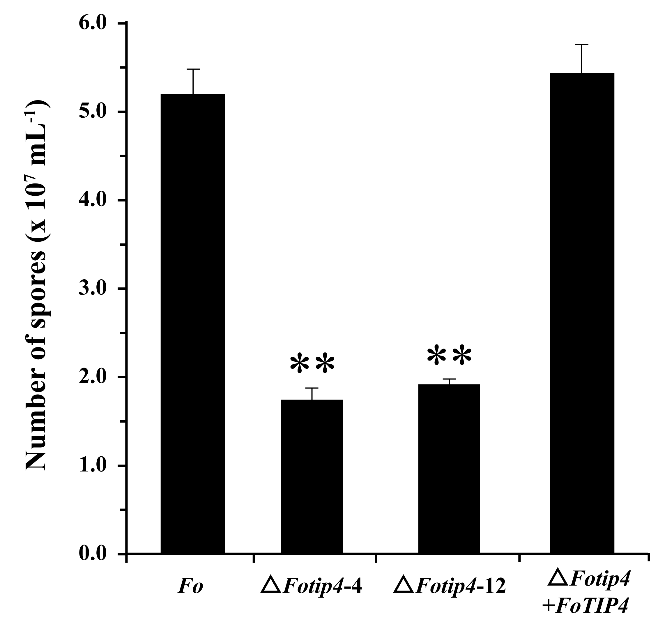


**Figure S8 Number of spores of *Fo*, Δ*Fotip4* mutants and complemented strain (Δ*Fotip4* + *FoTIP4*) for 5 days.** The data represent the mean ± SD of n = 3 independent experiments. Asterisks denote student’s *t* test signiﬁcant difference compared with *Fo* (**P < 0.01).
