# Supplementary material for: Target of rapamycin controls hyphal growth and pathogenicity through FoTIP4 in Fusarium oxysporum
Source: Mol Plant Pathol. 2021 Jul 20;22(10):1239–55. doi: 10.1111/mpp.13108 (PMC8435236; doi:10.1111/mpp.13108)
Supplement: Supplementary file 9 — FIGURE S9 Quantification of FoTIP4‐GFP fluorescence in the nucleus and cytoplasm. Hyphae carrying a GFP‐tagged FoTIP4 were grown for 3 days in potato dextrose broth, RAP (1 μM) was added, and fungi were incubated for 12 hr. Data are presented as the mean ± SD of n = 2 independent experiments. **P < 0.01 compared with the DMSO group (Student’s t‐test) [file MPP-22-1239-s003.docx]

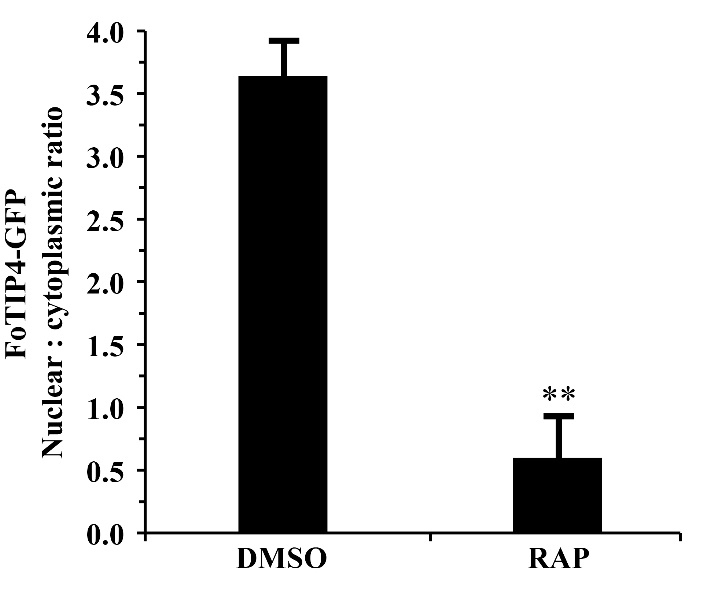


**Figure S9 Quantification of FoTIP4-GFP in nucleus and cytoplasm.** Hyphae carrying a GFP-tagged FoTIP4 were grown for 3 days in PDB medium, and the indicated drug RAP (1 μM) was added for 12 h. Data represent the mean ± SD of n = 2 independent experiments. Asterisks denote student’s *t* test signiﬁcant difference compared with DMSO (**P < 0.01).
