# Supplementary material for: Target of rapamycin controls hyphal growth and pathogenicity through FoTIP4 in Fusarium oxysporum
Source: Mol Plant Pathol. 2021 Jul 20;22(10):1239–55. doi: 10.1111/mpp.13108 (PMC8435236; doi:10.1111/mpp.13108)
Supplement: Supplementary file 10 — TABLE S1TOR genes in formae speciales of Fusarium oxysporum [file MPP-22-1239-s005.docx]

**Table S1** ***TOR* genes in forma specialis of *F. oxysporum*.**

| *TOR* | Forma specialis |
| --- | --- |
| *FOXG_18412* (*FoTOR1*) | Fusarium oxysporum f. sp. lycopersici (4287) |
| *FOXG_15946* (*FoTOR2*) |  |
| *FOIG_01765* | Fusarium oxysporum f. sp. cubense tropical race 4 54006 |
| *FOC1_g10015220* | Fusarium oxysporum f. sp. cubense race 1 |
| *FOCG_13386* | Fusarium oxysporum f. sp. radicis-lycopersici 26381 |
| *FOYG_09228* | Fusarium oxysporum FOSC 3-a |
| *FOZG_09320* | Fusarium oxysporum Fo47 |
| *FOWG_14365* | Fusarium oxysporum f. sp. lycopersici MN25 |
| *FOQG_16328* | Fusarium oxysporum f. sp. raphani 54005 |
| *FOQG_10061* |  |
| *FOPG_17299* | Fusarium oxysporum f. sp. conglutinans race 2 54008 |
| *FOPG_09340* |  |
| *FOMG_10886* | Fusarium oxysporum f. sp. melonis 26406 |
| *FOMG_16915* |  |
| *FOTG_16338* | Fusarium oxysporum f. sp. vasinfectum 25433 |
| *FOTG_17386* |  |
| *FOXB_12290* | Fusarium oxysporum Fo5176 |
| *FOXB_02566* |  |
| *FOVG_09941* | Fusarium oxysporum f. sp. pisi HDV247 |
| *FOVG_19124* |  |
| *FOVG_18014* |  |
| *AU210_012382* | Fusarium oxysporum f. sp. radicis-cucumerinum |
| *AU210_008856* |  |
