# Supplementary material for: Target of rapamycin controls hyphal growth and pathogenicity through FoTIP4 in Fusarium oxysporum
Source: Mol Plant Pathol. 2021 Jul 20;22(10):1239–55. doi: 10.1111/mpp.13108 (PMC8435236; doi:10.1111/mpp.13108)
Supplement: Supplementary file 14 — TABLE S5 Probe sequences of FOXG_01365 and FOXG_13331 promoters [file MPP-22-1239-s011.docx]

**Table S5 Probe sequences of *FOXG_01365* and *FOXG_13331* promoters.**

| Probe | Sequence |
| --- | --- |
| *PFOXG_01365* probe | GCCAAACAAGAGTAGTTGAGATGAGGTATGCCAGAATG |
| *PFOXG_01365* mutant probe | GCCAAACAAGAGTAGTTAAAAAAAAAAATGCCAGAATG |
| *PFOXG_13331* probe | CGAATAGAGGTGATGAGCTGGGCCAAACCGTGCCCGTGGGGCTCAATTGTTGGTGTAGCAGCCATTTCCTGGAAGTAGATATCACTCAAGTGTACACCACGCGAATTTTTCTTTGAACG |
| *PFOXG_13331* mutant probe | CGAATAGAGAAAAAAAAAAGGGCCAAACCGTGCCCGTGGGGCTCAATTGTTGGTGTAGCAGCCATTTCCTGGAAGTAGATATCACTCAAGTGTACACCACGCGAAAAAAAAATTGAACG |
