# Supplementary material for: Target of rapamycin controls hyphal growth and pathogenicity through FoTIP4 in Fusarium oxysporum
Source: Mol Plant Pathol. 2021 Jul 20;22(10):1239–55. doi: 10.1111/mpp.13108 (PMC8435236; doi:10.1111/mpp.13108)
Supplement: Supplementary file 15 — TABLE S6 Primers used for cloning and reverse transcription PCR in this study [file MPP-22-1239-s010.docx]

**Table S6** **Primers used for genes cloning and RT-PCR in this study.**

| Primer name | Primer sequence (5’-3’) |
| --- | --- |
| Deletion primers | |
| FoTOR1 5’-U F | GGTACCCCGGTGCTATGCACCTCTC |
| FoTOR1 5’-U R | CTTCAATATCAGTTAACGTCGCGGCCGCGTATTTGCTGAGCGTCTG |
| FoTOR1-Hph-F | CAGACGCTCAGCAAATACGCGGCCGCGACGTTAACTGATATTGAAG |
| FoTOR1-Hph-R | CTCTTCTTCTGCAGAATCTATTCCTTTGCCCTCGGACG |
| FoTOR1 3’-D F | CGTCCGAGGGCAAAGGAATAGATTCTGCAGAAGAAGAG |
| FoTOR1 3’-D R | TTAATTAAGTTCTGAAGGAGGATGGGGAG |
| FoTOR2 5’-U F | GGTACCGCGATCGCATCAAGGGCAGGAAC |
| FoTOR2 5’-U R | CTTCAATATCAGTTAACGTCGCGGCCGCGGGGTAGCCTCAGCCG |
| FoTOR2-Hph-F | CGGCTGAGGCTACCCCGCGGCCGCGACGTTAACTGATATTGAAG |
| FoTOR2-Hph-R | CTGCTGCACACGATCCAGCGCCTATTCCTTTGCCCTCGGACG |
| FoTOR2 3’-D F | CGTCCGAGGGCAAAGGAATAGGCGCTGGATCGTGTGCAGCAG |
| FoTOR2 3’-D R | TTAATTAATCTTTAAATAGTTAATAATAGC |
| FoFKBP12 5’-U F | GGTACCCGAATCTTACACTACATACAAC |
| FoFKBP12 5’-U R | GTTCAGGCTTTTTCATCTTGAAAAAGAGGAG |
| FoFKBP12-Hph-F | CTCCTCTTTTTCAAGATGAAAAAGCCTGAAC |
| FoFKBP12-Hph-R | AGATAGTTGAGGGACCTATTCCTTTGCCCT |
| FoFKBP12 3’-D F | AGGGCAAAGGAATAGGTCCCTCAACTATCT |
| FoFKBP12 3’-D R | TTAATTAAGTTGAGTGGAACAAGAGAGCGCG |
| FoTIP4 5’-U F | GGTACCGTAGTGCTTTCGCTGGCACTGCCTGTCAGATCC |
| FoTIP4 5’-U R | GTTCAGGCTTTTTCATGGTGGCGGCCGCGTCGAATGAAATATGAATGG |
| FoTIP4-Hph-F | CCATTCATATTTCATTCGACGCGGCCGCCACCATGAAAAAGCCTGAAC |
| FoTIP4-Hph-R | GGAAAGAGTCGAGAGTTTCCTGCAGGCTATTCCTTTGCCCTCGG |
| FoTIP4 3’-D F | CCGAGGGCAAAGGAATAGCCTGCAGGAAACTCTCGACTCTTTCC |
| FoTIP4 3’-D R | TTAATTAACGCAAATTTGTCTCCCCAGCCCTCG |
|  |  |
| Cloning primers | |
| PFoFKBP12 F | GCGATCGCCGAATCTTACACTACATACAAC |
| PFoFKBP12 R | GCGGCCGCCTTGAAAAAGAGGAG |
| FoFKBP12 F | GCGGCCGCATGGGTGTTCAGAAGACCAT |
| FoFKBP12 R | CCTGCAGGGTTAATCTTCTGGAGCTCAAC |
| PFoTIP4 F | GCGATCGCAACAAATAAGGTGTCACATATC |
| PFoTIP4 R | GCGGCCGCGTCGAATGAAATATGAATG |
| FoTIP4 F | GCGGCCGCATGGGGAAGTTGTTCGGCAG |
| FoTIP4 R | CCTGCAGGCAGCAGTGCACTGGATTCAGC |
|  |  |
| RT-PCR primers | |
| RT FoEIF1α F | CGGTACTGGTGAGTTCGAGGCT |
| RT FoEIF1α R | TGTTGATGGCGACAATGAGGTT |
| RT FoTIP4 F | CCTTTCGGAACTCACCAGTC |
| RT FoTIP4 R | TGTGGGCTTCCTCGTAATGT |
| RT FoTOR1 F | CTGCCCGTCAATAAGAAGGGT |
| RT FoTOR1 R | GTTACCAGCTTGGGCAGCG |
| RT FoTOR2 F | TTGCCTGCTAACAAGAAGGCC |
| RT FoTOR2 R | ACTACCAGCTTGTGCATTC |
| RT FoFKBP12 F | CCGTGGTGCCTTTGTGGTCC |
| RT FoFKBP12 R | TCGCCGAGCTTCATTTGGGT |
| RT FOXG_13684 F | CTCGTTCACCGCAAACATCAGT |
| RT FOXG_13684 R | CGCCATAATACGCCAGCATCTC |
| RT FOXG_04931 F | GCACAACTGGGCAATGATGACA |
| RT FOXG_04931 R | ACTGCCTCGCCTTCTTGAGTGT |
| RT FOXG_05170 F | TGGTGAGGTTACTGGTGCTGAG |
| RT FOXG_05170 R | ATCGGAGACGTTGAGGTTGTTC |
| RT FOXG_01269 F | CTGGCACCGACAACACCAACCC |
| RT FOXG_01269 R | TTCCGCCCTTGGAGGCAATCTC |
| RT FOXG_02212 F | TTGTGCGAAACGATGCCAGAG |
| RT FOXG_02212 R | GTCCACTTCAGCTTGCGAGGG |
| RT FOXG_00864 F | GTTGGTGGTGCCCGTGGTTCA |
| RT FOXG_00864 R | GCTCTGCGGTGCCAATGAAAG |
| RT FOXG_11242 F | TGAGGCGAAAGACAGCACAGT |
| RT FOXG_11242 R | CGAAAGCAAGGCTTGTAGACC |
| RT FOXG_12883 F | GGATGCGGTGGGCCTGAAAT |
| RT FOXG_12883 R | CCTGAAAGGGCTGAAGTTGG |
| RT FOXG_17421 F | TATCCAAGCCTTGCACTCCT |
| RT FOXG_17421 R | GCTCGGCATAGCCAAATCAG |
| RT FOXG_01365 F | CTACGTCAAGCCACCTCACCC |
| RT FOXG_01365 R | TTGCCCTACCATTTGCTCCTG |
| RT FOXG_14550 F | ACCTGCTACTTATGATGCG |
| RT FOXG_14550 R | CAGCGTTATGAAGAACCAC |
| RT FOXG_13331 F | AATGCGGAGGCAAGAACTG |
| RT FOXG_13331 R | GGACATAGCGACGAACACG |
